# Supplementary material for: Form and function of actin impacts actin health and aging
Source: iScience. 2026 Aug 24;29(9):117256. doi: 10.1016/j.isci.2026.117256 (PMC13524715; doi:10.1016/j.isci.2026.117256)
Supplement: Supplementary file 1 [file mmc1.pdf]

iScience, Volume *29*

Supplemental information

Form and function of actin impacts

actin health and aging

Maxim Averbukh, Hunter T. Nelson, Tiffany Wang, Caitlin M. Lange, Athena Alcala, Juri Kim, Daniella Suh, Matthew Vega, Shihong Max Gao, Naibedya Dutta, Khristina E. Young, Rebecca Aviles Barahona, Aeowynn J. Coakley, Joe A. Gerke, Sofia F. Odron, Jacqueline G. Clarke, Navjot Singh, Matthew S. Nickel, Jacob C. Peterson, Hannah L. Lam-Truong, Darius Moaddeli, Toni Castro Torres, Maria Oorloff, Melissa Peruch, Tripti Nair, Hemal H. Mehta, Max A. Thorwald, Shoichiro Ono, Sean P. Curran, Meng C. Wang, Bérénice A. Benayoun, Cristal M. Hill, Tyler A. Johnson, Eileen M. Crimmins, Thalida Em Arpawong, Caroline Kumsta, Gilberto Garcia, and Ryo Higuchi-Sanabria

Supplemental Figures and Figure Legends

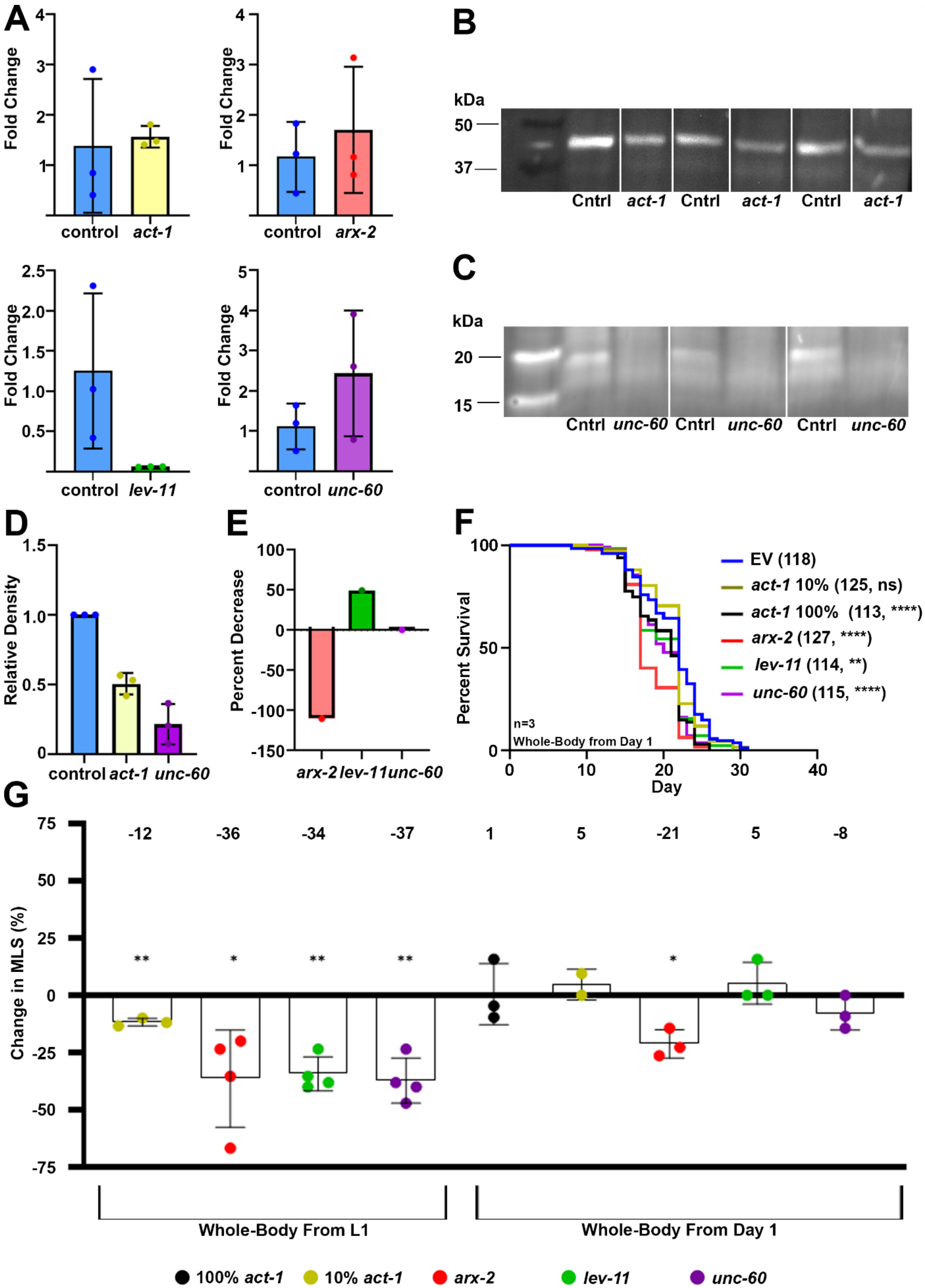

A
Fold Change
control
act-1
arx-2
lev-11
unc-60
B
kDa
50
37
Cntrl
act-1
C
20
15
D
Relative Density
E
Percent Decrease
F
Percent Survival
Day
EV (118)
act-1 10% (125, ns)
act-1 100% (113, ****)
arx-2 (127, ****)
lev-11 (114, **)
unc-60 (115, ****)
n=3
Whole-Body from Day 1
G
Change in MLS (%)
-12
-36
-34
-37
1
5
-21
5
-8
Whole-Body From L1
Whole-Body From Day 1
100% act-1
10% act-1
arx-2
lev-11
unc-60

Fig. S1. Knockdown of actin and ARPs reduce lifespan. (A) RT-qPCR of wild-type Day 1 adult worms raised on either empty vector (control, blue), 10% *act-1* (yellow), *arx-2* (red), *lev-11* (green), or *unc-60* (purple) RNAi. n=3. Represented as fold change compared to house-keeping genes. **(B)** Western blots of wild-type Day 1 adult worms raised on empty vector or 10% *act-1* RNAi. Membranes were probed with an anti-actin antibody (proteintech, Cat #66009). n=3. **(C)** Western blots of wild-type Day 1 adult worms raised on empty vector or *unc-60* RNAi. Membranes were probed with an anti-UNC-60 antibody[25]. n=3. **(D)** Quantification of western blots represented as relative density compared to the control, where control relative density is set at 1. **(E)** Quantification of the proportion of bulk RNA-seq reads mapped to regions of *arx-2* (red), *lev-11* (green), and *unc-60* (purple) that lie outside the respective RNAi sequence overlap compared to empty vector control. **(F)** Lifespans of wild-type N2 animals grown on empty vector (EV, blue), 100% *act-1* (black), 10% *act-1* (yellow), *arx-2* (red), *lev-11* (green), or *unc-60* (purple) RNAi from Day 1 of adulthood. See **Table S1** for lifespan statistics. **(G)** Average lifespan change (% MLS change) in wild-type animals (grown from L1 or D1 on RNAi) compared to control. Horizontal values at the top represent the average percent change in median lifespan for each condition. Error bars indicate the s.d. * = $p < 0.05$, ** = $p < 0.01$ by a two-sided, one-sample t-test compared to hypothetical mean of 0.

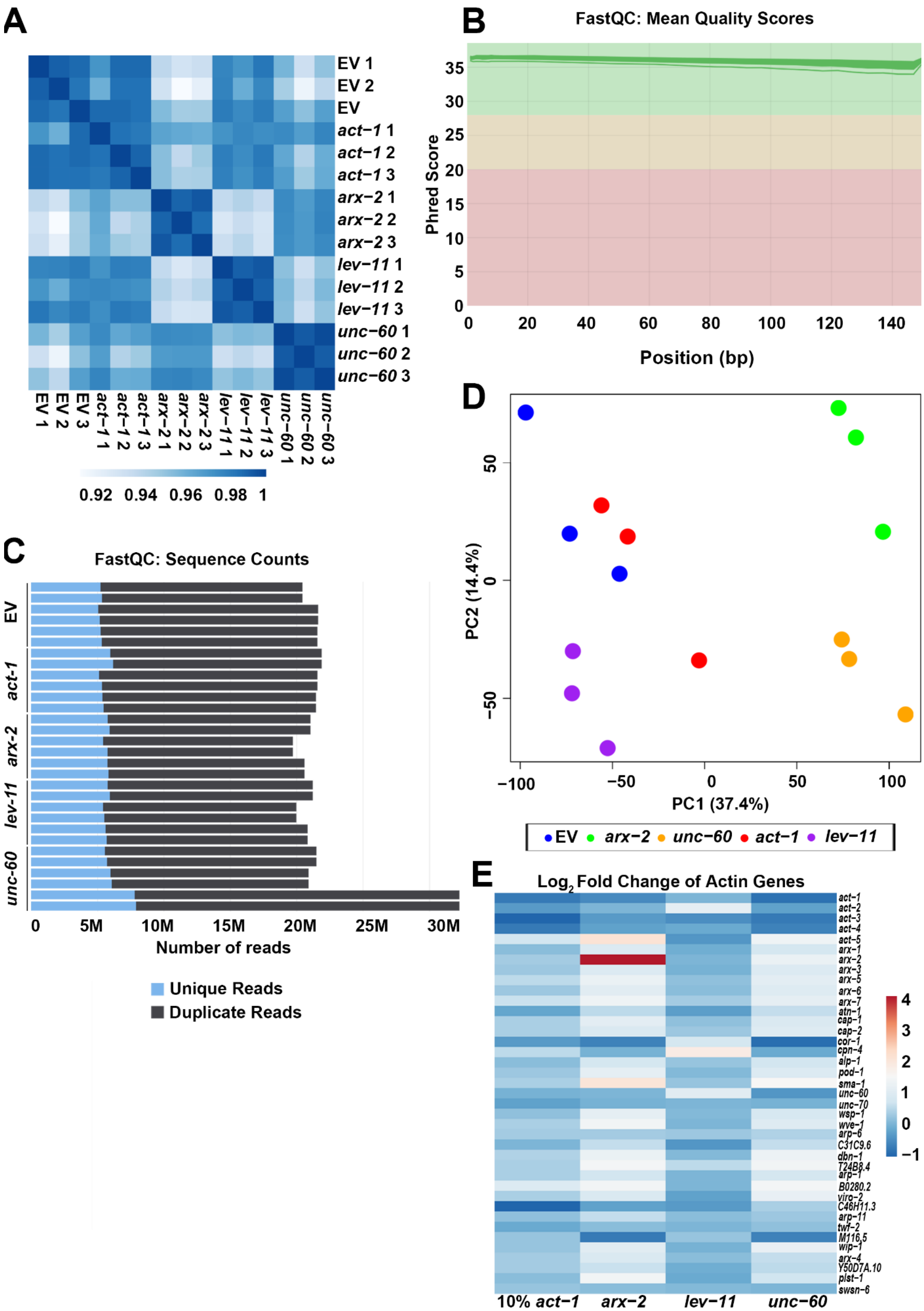
A
EV 1
EV 2
EV
act−1 1
act−1 2
act−1 3
arx−2 1
arx−2 2
arx−2 3
lev−11 1
lev−11 2
lev−11 3
unc−60 1
unc−60 2
unc−60 3
EV 1
EV 2
EV 3
act−1 1
act−1 2
act−1 3
arx−2 1
arx−2 2
arx−2 3
lev−11 1
lev−11 2
lev−11 3
unc−60 1
unc−60 2
unc−60 3
0.92 0.94 0.96 0.98 1
B
FastQC: Mean Quality Scores
Phred Score
0 5 10 15 20 25 30 35
0 20 40 60 80 100 120 140
Position (bp)
C
FastQC: Sequence Counts
EV
act-1
arx-2
lev-11
unc-60
0 5M 10M 15M 20M 25M 30M
Number of reads
Unique Reads
Duplicate Reads
D
PC2 (14.4%)
50 0 −50
−100 −50 0 50 100
PC1 (37.4%)
EV arx−2 unc−60 act−1 lev−11
E
Log2 Fold Change of Actin Genes
act−1
act−2
act−3
act−4
act−5
arx−1
arx−2
arx−3
arx−5
arx−6
arx−7
atn−1
cap−1
cap−2
cor−1
cpn−4
alp−1
pod−1
sma−1
unc−60
unc−70
wsp−1
wve−1
arp−6
C31C9.6
dbn−1
T24B8.4
arp−1
B0280.2
viro−2
C46H11.3
arp−11
twf−2
M116.5
wip−1
arx−4
Y50D7A.10
plst−1
swsn−6
4 3 2 1 0 −1
10% act−1
arx−2
lev−11
unc−60

Fig. S2: Quality control for RNA-seq libraries of *C. elegans* exposed to actin and ABP knockdown. (A) Spearman correlation plot of all RNA sequencing libraries. **(B)** Mean quality score (Phred score) of each RNA sequencing library. X-axis and Y-axis indicate the base pair position of each sequence and the Phred score, respectively. The graph was generated by MultiQC tool[199]. **(C)** The number of unique (blue) and duplicated (grey) reads from each pair-wise sequencing library (n=3). **(D)** PCA plots of actin and ABP knockdown in *C. elegans*. **(E)** Heatmap of log2(fold changes) for all genes annotated as cytoskeleton: Actin function in WormCat[200]. See **Table S6** for expression details of the genes used in the heatmap.

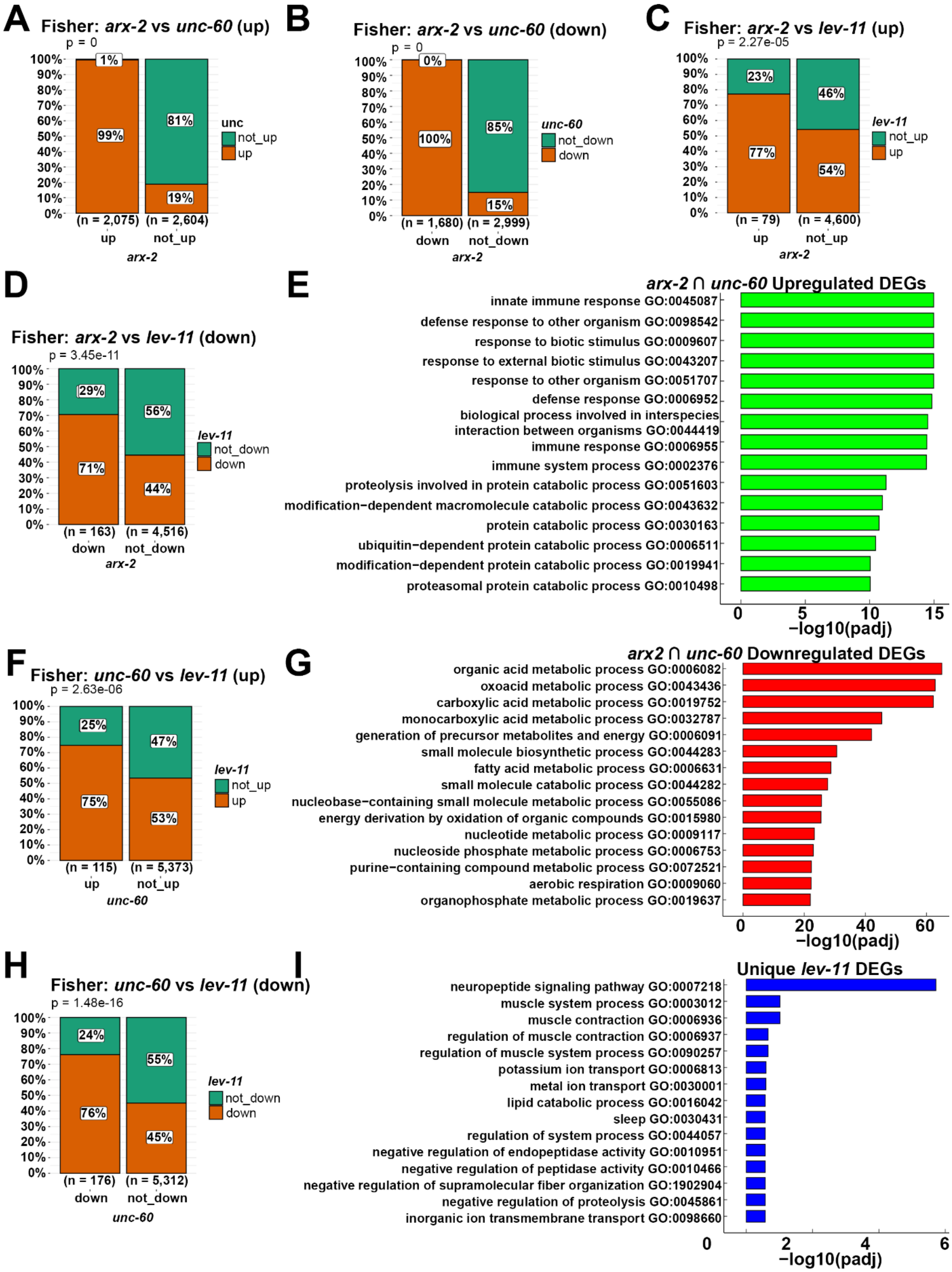
A
Fisher: arx-2 vs unc-60 (up)
p = 0
1%
99%
81%
19%
unc
not_up
up
(n = 2,075)
(n = 2,604)
up
not_up
arx-2
B
Fisher: arx-2 vs unc-60 (down)
p = 0
0%
100%
85%
15%
unc-60
not_down
down
(n = 1,680)
(n = 2,999)
down
not_down
arx-2
C
Fisher: arx-2 vs lev-11 (up)
p = 2.27e-05
23%
77%
46%
54%
lev-11
not_up
up
(n = 79)
(n = 4,600)
up
not_up
arx-2
D
Fisher: arx-2 vs lev-11 (down)
p = 3.45e-11
29%
71%
56%
44%
lev-11
not_down
down
(n = 163)
(n = 4,516)
down
not_down
arx-2
E
arx-2 ∩ unc-60 Upregulated DEGs
innate immune response GO:0045087
defense response to other organism GO:0098542
response to biotic stimulus GO:0009607
response to external biotic stimulus GO:0043207
response to other organism GO:0051707
defense response GO:0006952
biological process involved in interspecies interaction between organisms GO:0044419
immune response GO:0006955
immune system process GO:0002376
proteolysis involved in protein catabolic process GO:0051603
modification−dependent macromolecule catabolic process GO:0043632
protein catabolic process GO:0030163
ubiquitin−dependent protein catabolic process GO:0006511
modification−dependent protein catabolic process GO:0019941
proteasomal protein catabolic process GO:0010498
−log10(padj)
F
Fisher: unc-60 vs lev-11 (up)
p = 2.63e-06
25%
75%
47%
53%
lev-11
not_up
up
(n = 115)
(n = 5,373)
up
not_up
unc-60
G
arx2 ∩ unc-60 Downregulated DEGs
organic acid metabolic process GO:0006082
oxoacid metabolic process GO:0043436
carboxylic acid metabolic process GO:0019752
monocarboxylic acid metabolic process GO:0032787
generation of precursor metabolites and energy GO:0006091
small molecule biosynthetic process GO:0044283
fatty acid metabolic process GO:0006631
small molecule catabolic process GO:0044282
nucleobase−containing small molecule metabolic process GO:0055086
energy derivation by oxidation of organic compounds GO:0015980
nucleotide metabolic process GO:0009117
nucleoside phosphate metabolic process GO:0006753
purine−containing compound metabolic process GO:0072521
aerobic respiration GO:0009060
organophosphate metabolic process GO:0019637
−log10(padj)
H
Fisher: unc-60 vs lev-11 (down)
p = 1.48e-16
24%
76%
55%
45%
lev-11
not_down
down
(n = 176)
(n = 5,312)
down
not_down
unc-60
I
Unique lev-11 DEGs
neuropeptide signaling pathway GO:0007218
muscle system process GO:0003012
muscle contraction GO:0006936
regulation of muscle contraction GO:0006937
regulation of muscle system process GO:0090257
potassium ion transport GO:0006813
metal ion transport GO:0030001
lipid catabolic process GO:0016042
sleep GO:0030431
regulation of system process GO:0044057
negative regulation of endopeptidase activity GO:0010951
negative regulation of peptidase activity GO:0010466
negative regulation of supramolecular fiber organization GO:1902904
negative regulation of proteolysis GO:0045861
inorganic ion transmembrane transport GO:0098660
−log10(padj)

Fig. S3: Knockdown of various ABP results in highly concordant transcriptomic changes. Bar plots to visualize the overlap of up- and down-regulated differentially expressed genes (DEGs) between animals treated with **(A)(B)** *arx-2* and *unc-60*, **(C)(D)** *arx-2* and *lev-11*, and **(F)(H)** *unc-60* and *lev-11* using ggbarstats function in R. Statistical significance of the overlap was assessed using Fisher's exact test. GO analysis of DEGs **(E)** upregulated and **(G)** downregulated exclusively in both *arx-2* and *unc-60* conditions. **(I)** GO analysis of DEGs unique to the *lev-11* condition. See **Table S7** for Go IDs, genes and statistics of the GO Analysis.

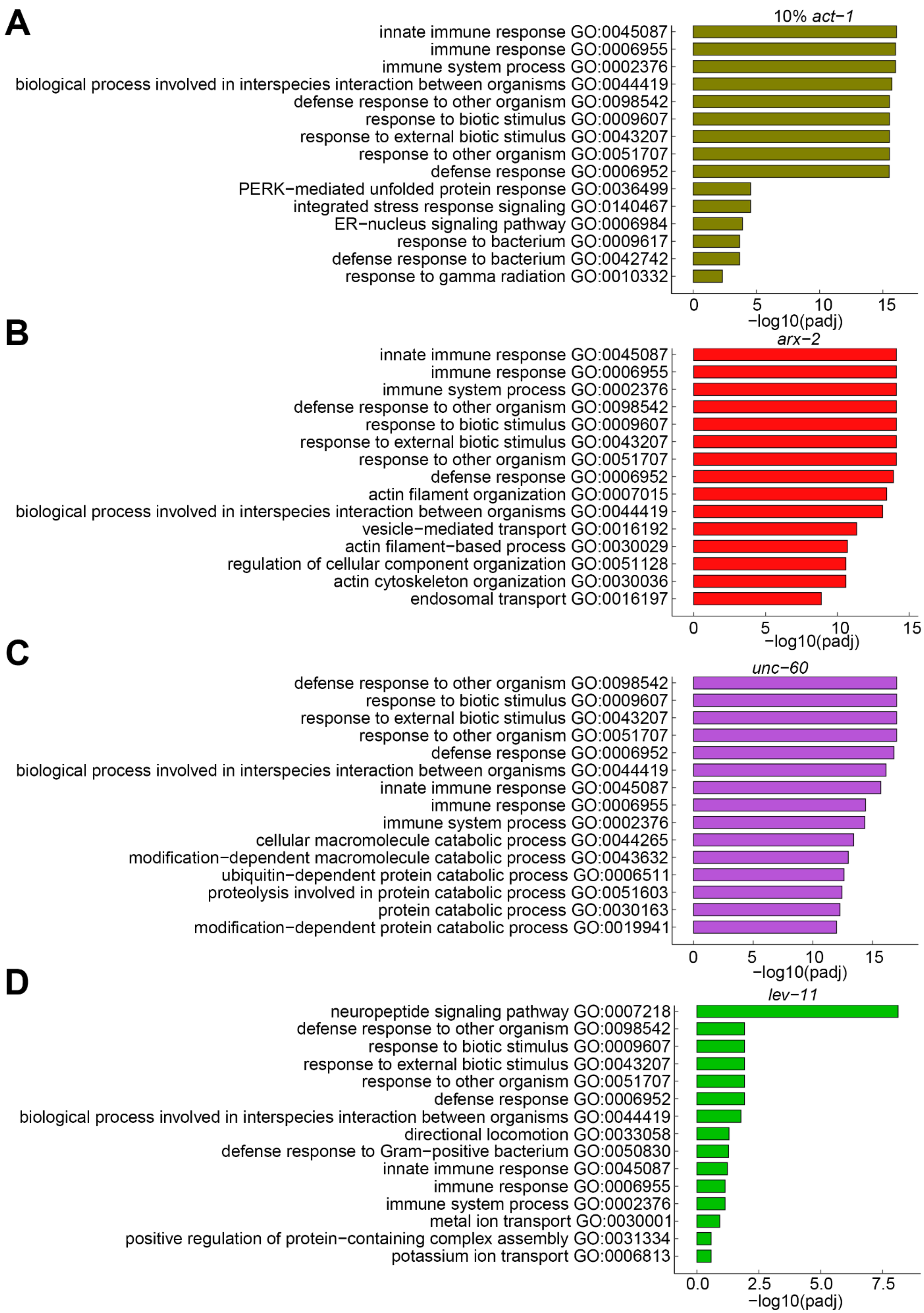
A
10% act−1
innate immune response GO:0045087
immune response GO:0006955
immune system process GO:0002376
biological process involved in interspecies interaction between organisms GO:0044419
defense response to other organism GO:0098542
response to biotic stimulus GO:0009607
response to external biotic stimulus GO:0043207
response to other organism GO:0051707
defense response GO:0006952
PERK−mediated unfolded protein response GO:0036499
integrated stress response signaling GO:0140467
ER−nucleus signaling pathway GO:0006984
response to bacterium GO:0009617
defense response to bacterium GO:0042742
response to gamma radiation GO:0010332
0
5
10
15
−log10(padj)
B
arx−2
innate immune response GO:0045087
immune response GO:0006955
immune system process GO:0002376
defense response to other organism GO:0098542
response to biotic stimulus GO:0009607
response to external biotic stimulus GO:0043207
response to other organism GO:0051707
defense response GO:0006952
actin filament organization GO:0007015
biological process involved in interspecies interaction between organisms GO:0044419
vesicle−mediated transport GO:0016192
actin filament−based process GO:0030029
regulation of cellular component organization GO:0051128
actin cytoskeleton organization GO:0030036
endosomal transport GO:0016197
0
5
10
15
−log10(padj)
C
unc−60
defense response to other organism GO:0098542
response to biotic stimulus GO:0009607
response to external biotic stimulus GO:0043207
response to other organism GO:0051707
defense response GO:0006952
biological process involved in interspecies interaction between organisms GO:0044419
innate immune response GO:0045087
immune response GO:0006955
immune system process GO:0002376
cellular macromolecule catabolic process GO:0044265
modification−dependent macromolecule catabolic process GO:0043632
ubiquitin−dependent protein catabolic process GO:0006511
proteolysis involved in protein catabolic process GO:0051603
protein catabolic process GO:0030163
modification−dependent protein catabolic process GO:0019941
0
5
10
15
−log10(padj)
D
lev−11
neuropeptide signaling pathway GO:0007218
defense response to other organism GO:0098542
response to biotic stimulus GO:0009607
response to external biotic stimulus GO:0043207
response to other organism GO:0051707
defense response GO:0006952
biological process involved in interspecies interaction between organisms GO:0044419
directional locomotion GO:0033058
defense response to Gram−positive bacterium GO:0050830
innate immune response GO:0045087
immune response GO:0006955
immune system process GO:0002376
metal ion transport GO:0030001
positive regulation of protein−containing complex assembly GO:0031334
potassium ion transport GO:0006813
0.0
2.5
5.0
7.5
−log10(padj)

Fig. S4. Gene ontology analysis of actin disruption. GO analysis of unique DEGs not shared with another condition for **(A)** *act-1,* **(B)** *arx-2***, (C**) *unc-60* and **(D)** *lev-11*. All DEGs were selected for adj p-val < 0.05, and the GOs were biological processes (BP) with q-value < 0.5. See **Table S7** for Go IDs, genes and statistics of the GO Analysis.

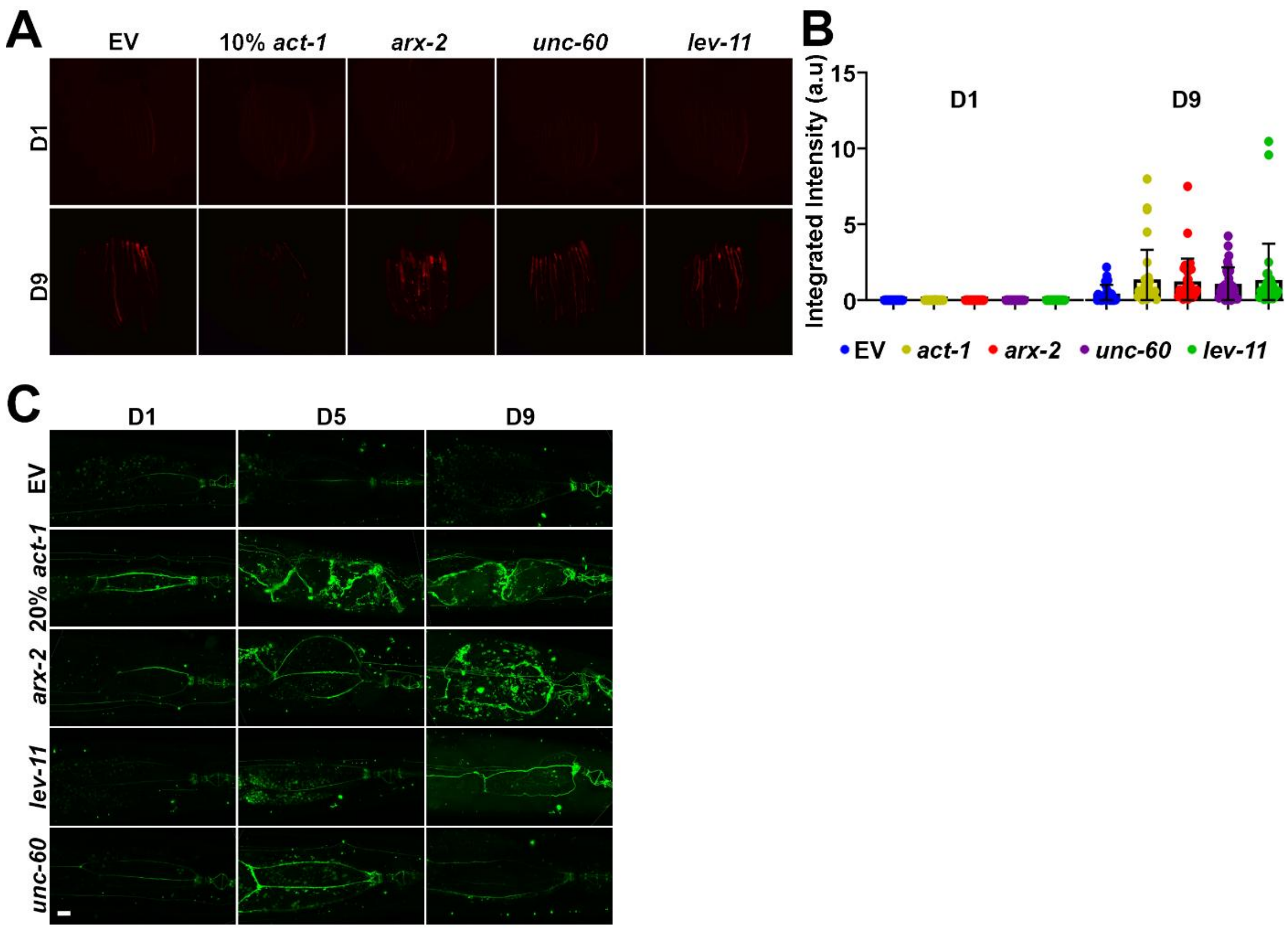

Fig. S5. Actin disruption leads to tissue-specific dysregulation of gut barrier integrity. (A) Representative stereoscope images of gut colonization at Day 1 and Day 9 in animals grown with EV, 10% *act-1*, *arx-2*, *lev-11*, or *unc-60* RNAi mixed with 20% HT115 bacteria driving mCherry expression. **(B)** Quantification of gut colonization fluorescence signal. 10-15 worms were imaged for two technical replicates for each of 3 independent biological replicates (with ≥ 150 worms total per replicate per strain/condition), analyzed via two-way ANOVA test. **(C)** Representative confocal images of DLG-1::GFP localization at Day 1, Day5, and Day 9 in animals grown with EV, 10% *act-1*, *arx-2*, *lev-11*, or *unc-60* RNAi.

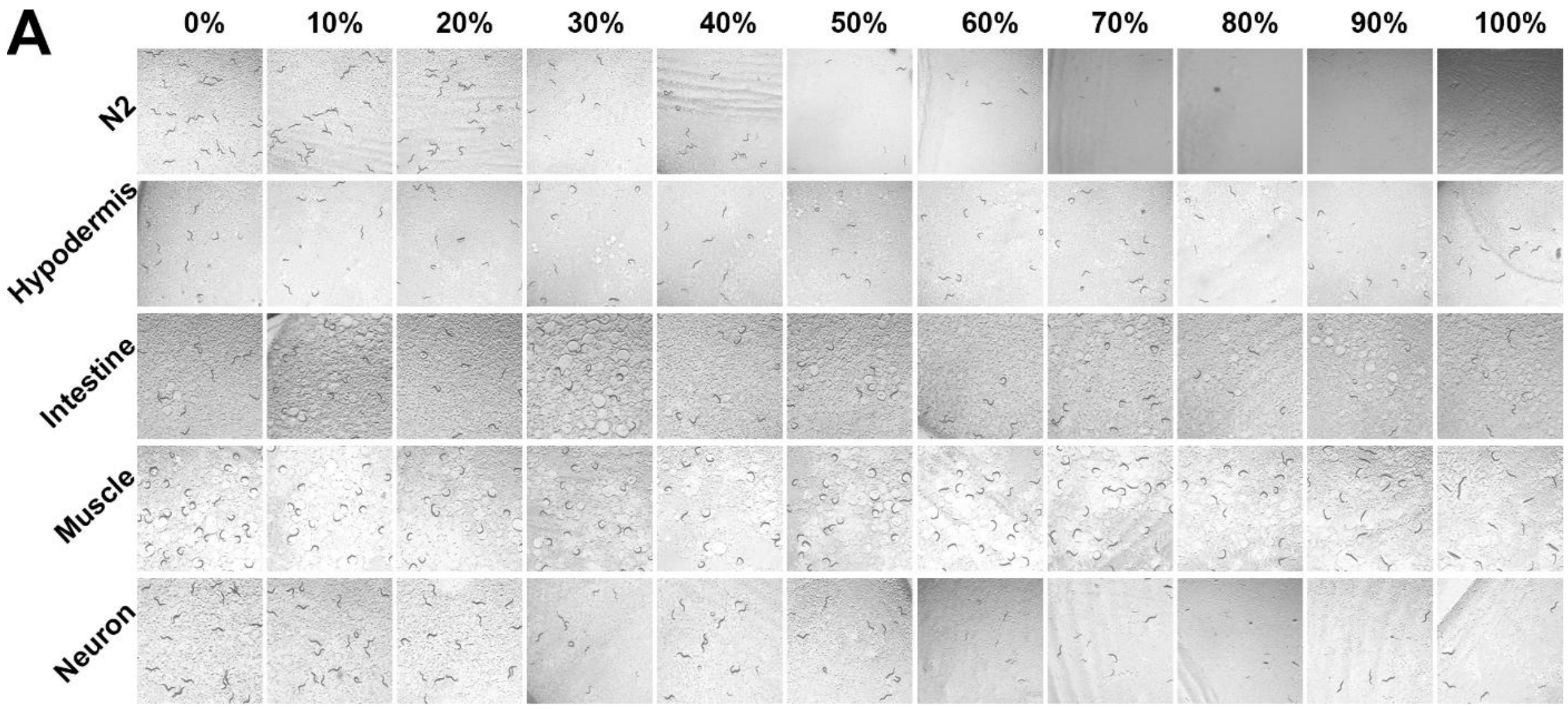

Fig. S6. Titration of RNAi for each tissue-specific RNAi strain. Representative stereoscopic images of *C. elegans* exposed to varying dilutions 0%-100% of actin RNAi from L1 stage of development. X-axis indicates dilution percentage with 0% being no actin RNAi and 100% meaning non-diluted RNAi. Y-axis represents the tissue where RNAi is functional with N2 being the wild-type control with whole-body functioning RNAi machinery.

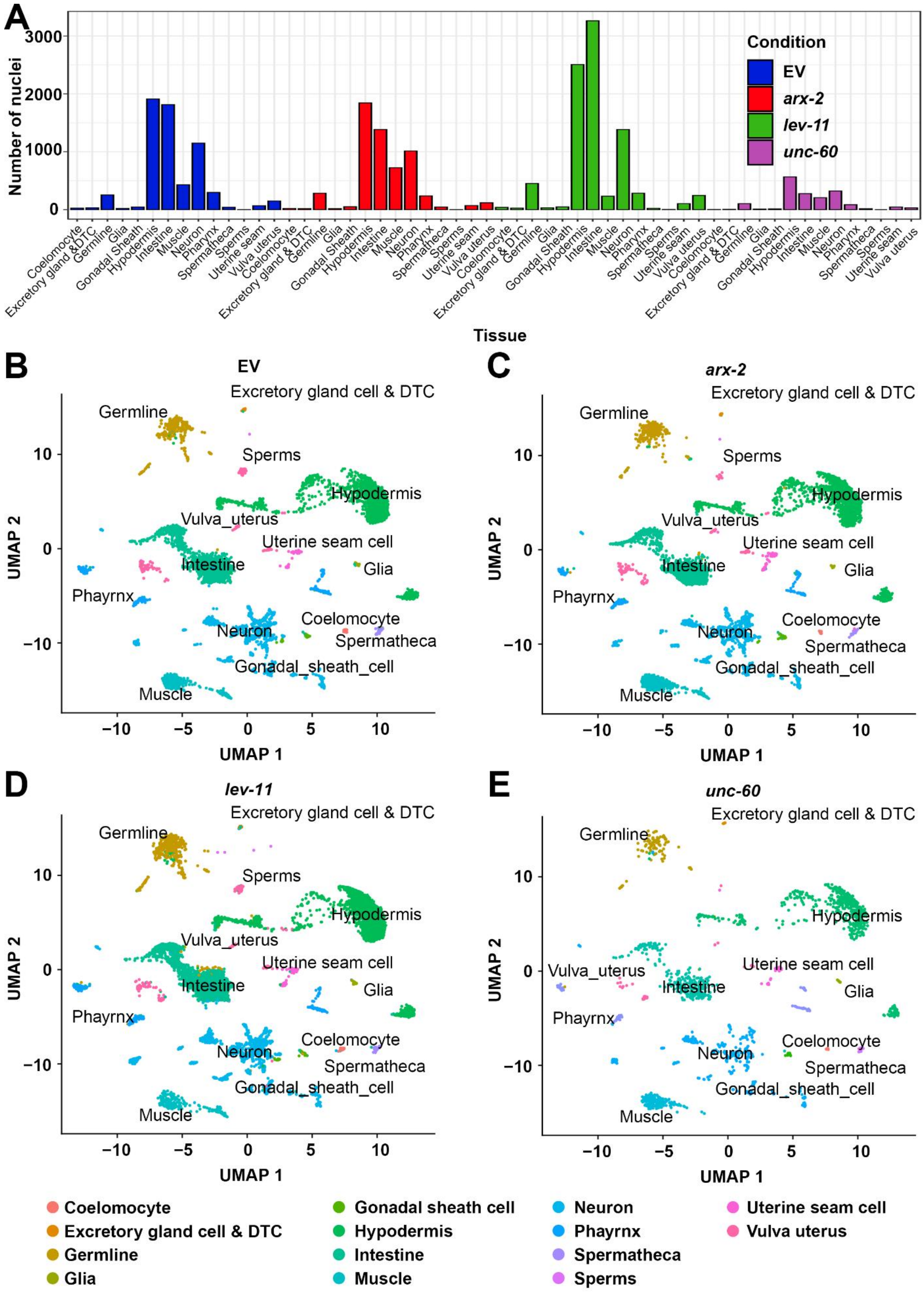

A
B
C
D
E
Condition
EV
arx-2
lev-11
unc-60
Number of nuclei
Tissue
Excretory gland cell & DTC
Germline
Sperms
Hypodermis
Vulva_uterus
Uterine seam cell
Intestine
Glia
Phayrnx
Neuron
Coelomocyte
Spermatheca
Gonadal_sheath_cell
Muscle
UMAP 1
UMAP 2
Coelomocyte
Excretory gland cell & DTC
Germline
Glia
Gonadal sheath cell
Hypodermis
Intestine
Muscle
Neuron
Phayrnx
Spermatheca
Sperms
Uterine seam cell
Vulva uterus

Fig. S7. Annotation and clustering of single-nuclei RNA sequencing. **(A)** Summary plot of all sequenced annotated nuclei for each tissue and condition. **(B-E)** UMAP of the data segmented to corresponding cell types, as identified by cell-type biomarkers in D1 **(B)** control animals and animals with **(C)** *arx-2*, **(D)** *lev-11*, and **(E)** *unc-60* knockdown.

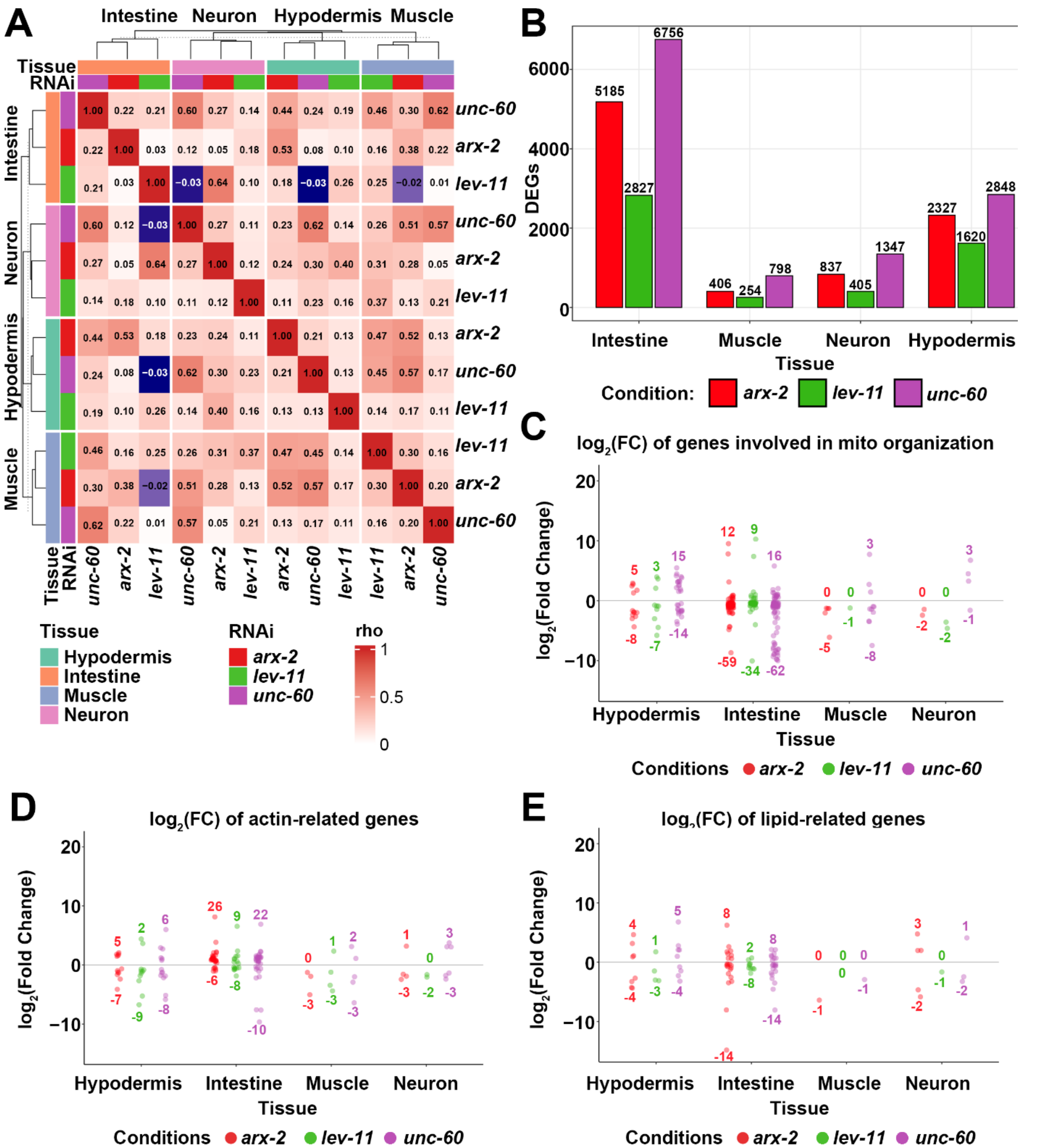

Fig. S8. Deconvolution of bulk RNA-seq analysis using a matched-reference snRNA-seq dataset. (A) Clustering of all samples based on the Spearman correlation between every tissue and condition. Clustering is performed within each tissue slice, and dendrograms illustrate the similarity structure. See **Table S8** for Spearman correlations between each sample. **(B)** Bar plot showing the number of DEGs for each condition and key selected tissues: Intestine, Muscle, Neuron and Hypodermis. (**C-E**) Dot plots showing significant DEGs involved in **(C)** mitochondrial organization (labeled as "involved in mitochondrion organization" in AmiGO2[198]), **(D)** actin maintenance (annotated as cytoskeleton: Actin function in WormCat) and **(E)** lipid regulation

(GO:0055088) under ABP knockdown in 4 key tissues: Hypodermis, Intestine, Muscle and Neuron. See **Table S9** for DEGs in each dotplot.

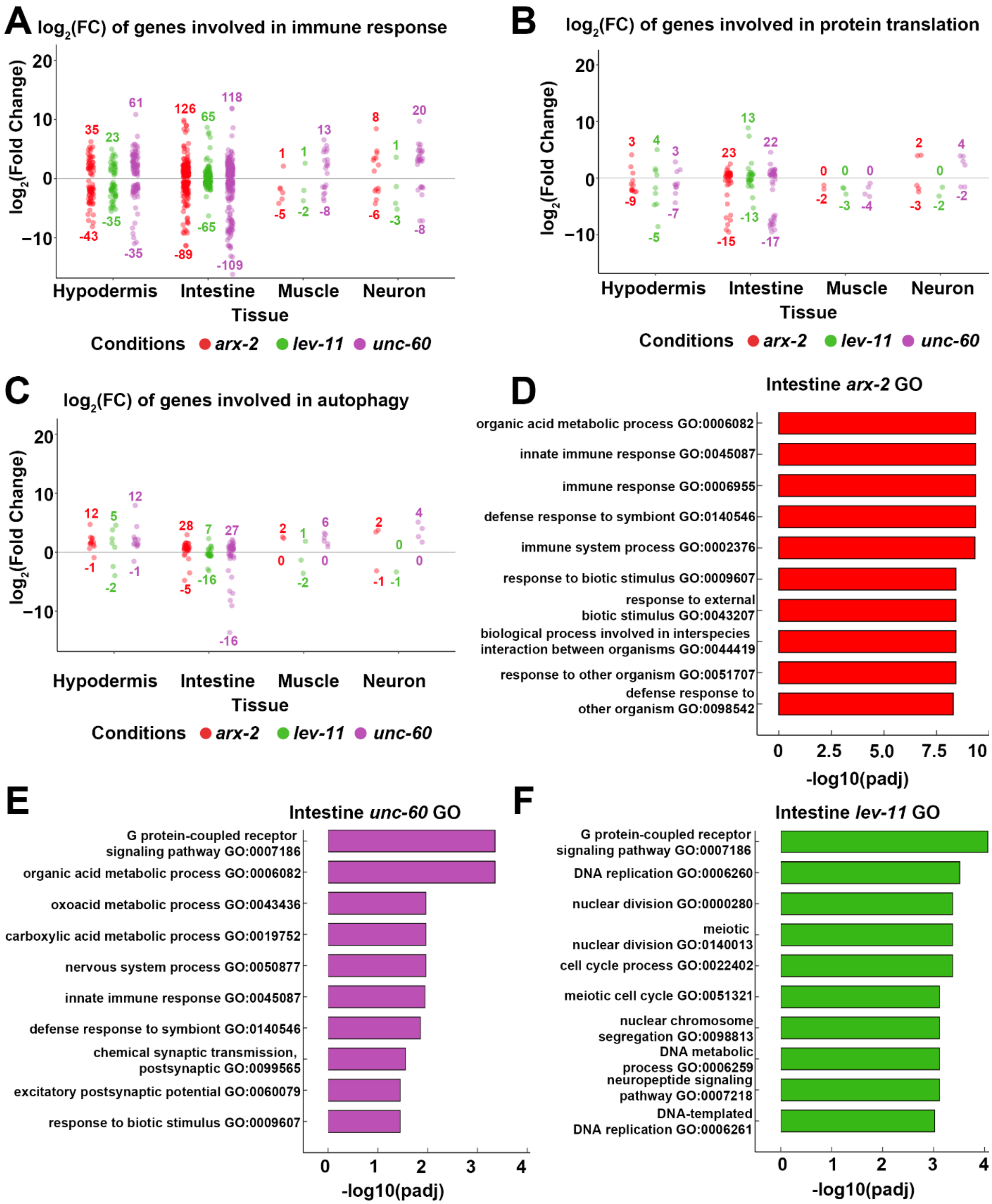

Fig. S9. RNAi of ABPs induced cell-type specific transcriptional responses. (A-C) Dot plots showing significant DEGs involved in **(A)** immune response (annotated as involved in "immune response" in AmiGO2[198]), **(B)** protein translation (directly annotated as "regulation of translation" in AmiGO2[198]) and **(C)** autophagy (directly annotated as "autophagy" in AmiGO2[198]) under ABP knockdown in 4 key tissues: Hypodermis, Intestine, Muscle and Neuron. See **Table**

S9 for DEGs in each dotplot. **(D-F)** GO analysis of DEGs in the intestine under ABP knockdown. See **Table S7** for Go IDs, genes and statistics of the GO Analysis.

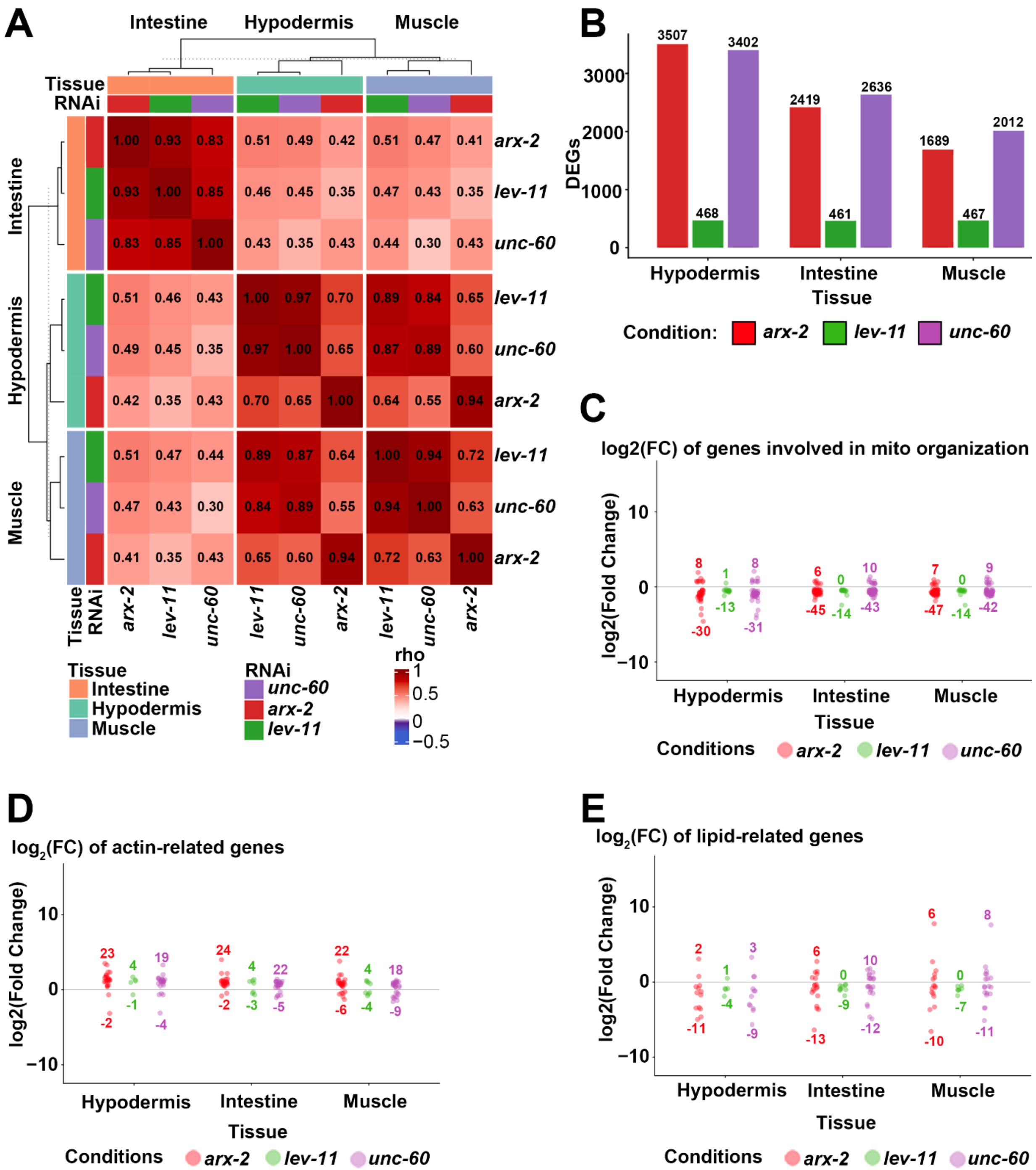

Fig. S10: Deconvolution of bulk RNA-seq analysis using a previously published N2 snRNA-seq dataset. (A) Clustering of all samples based on the Spearman correlation between every tissue and condition. Clustering is performed within each tissue slice, and dendrograms illustrate the similarity structure. See **Table S11** for Spearman correlations between each sample. **(B)** Bar plot showing the number of DEGs for each condition and key selected tissues: Hypodermis, Intestine, and Muscle. (**C-E**) Dot plots showing significant DEGs involved in **(C)** mitochondrial organization (labeled as "involved in mitochondrion organization" in AmiGO2[198]), **(D)** actin maintenance (annotated as cytoskeleton: Actin function in WormCat) and **(E)** lipid regulation (GO:0055088) under ABP knockdown in 4 key tissues: Hypodermis, Intestine, and Muscle. See **Table S9** for DEGs in each dotplot.

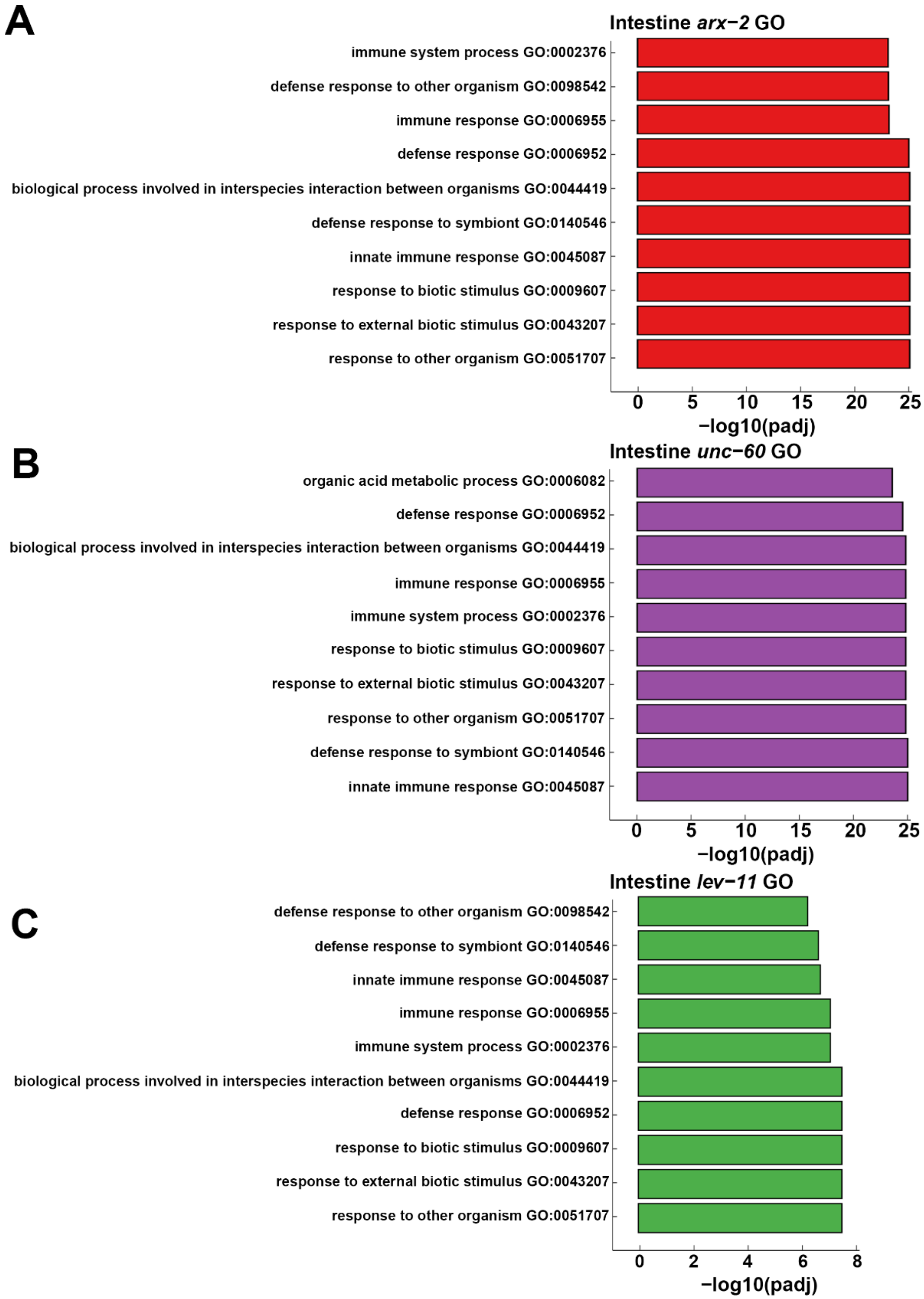

Fig. S11: Bulk RNA-seq data deconvolved against wild-type snRNA data shows parallel global transcriptional responses. (A-C) GO analysis of DEGs in the intestine under ABP knockdown. See **Table S10** for Go IDs, genes and statistics of the GO Analysis.

A

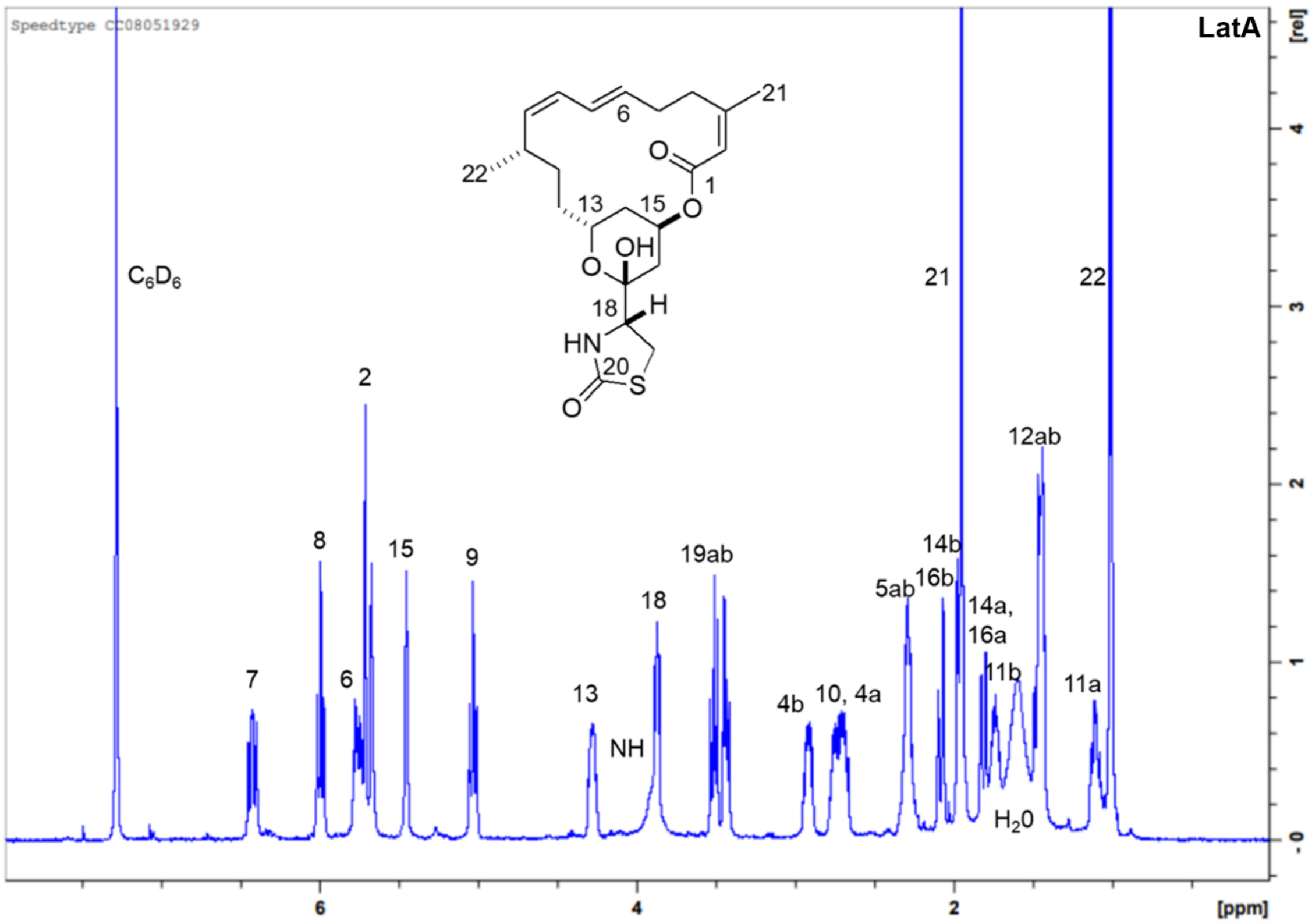
Speedtype CC08051929
LatA
[rel]
C6D6
21
22
2
8
15
9
7
6
13
NH
18
19ab
4b
10, 4a
5ab
14b
16b
14a,
16a
11b
12ab
11a
H20
[ppm]
HN
OH
O
S
H

B

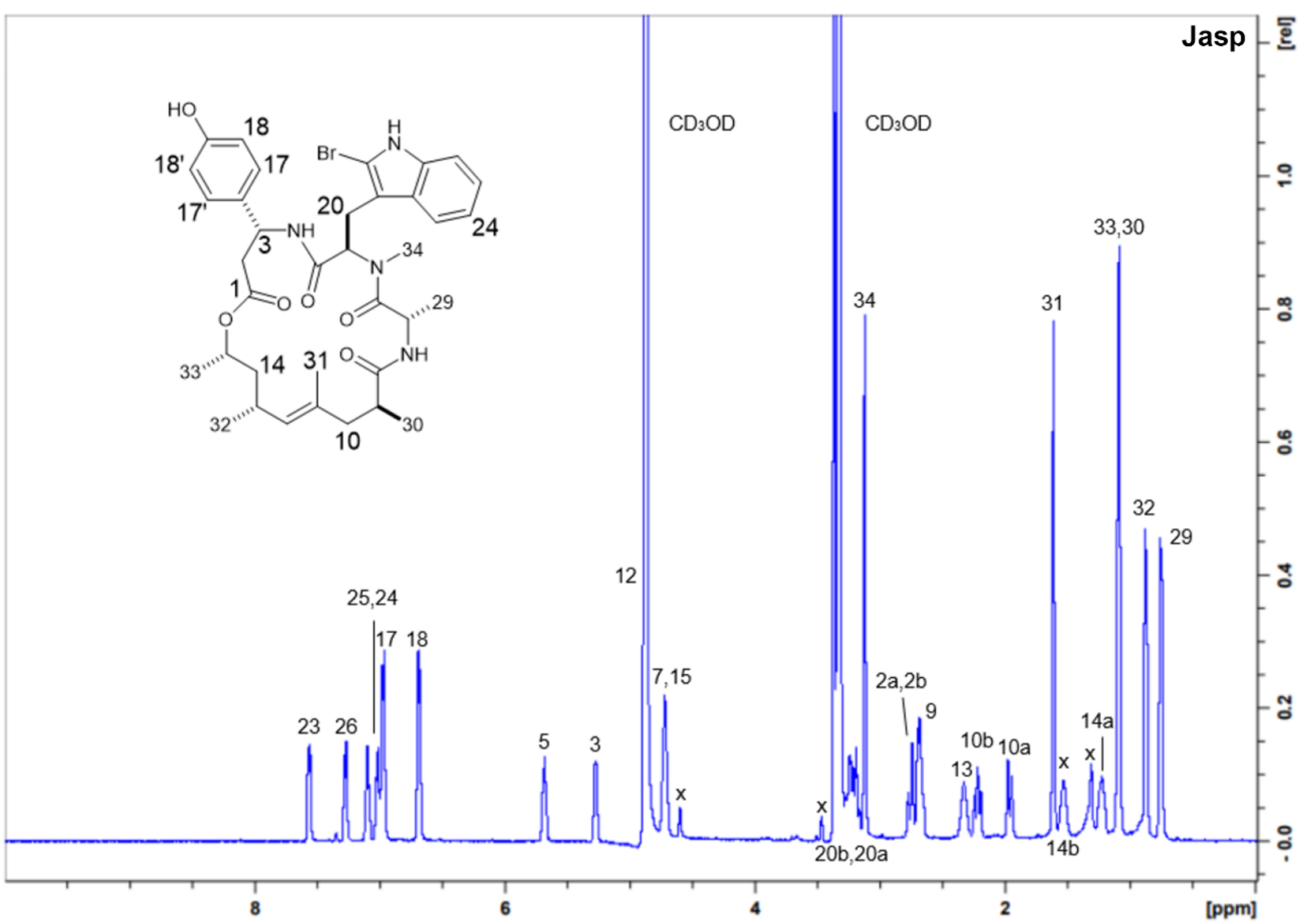
Jasp
[rel]
CD3OD
CD3OD
HO
Br
NH
N
34
29
33
14
31
32
30
10
1
3
17
17'
18
18'
20
24
12
25,24
23
26
17
18
5
3
7,15
x
x
20b,20a
34
2a,2b
9
13
10b
10a
31
x
x
14a
14b
33,30
32
29
[ppm]

Fig. S12. NMR analysis of LatA and Jasp. ^{1}H NMR spectrum of **(A)** latrunculin A (LatA) in Benzene-*d6* at 500 MHz and **(B)** jasplakinolide (Jasp) in CD_3OD at 500MHz

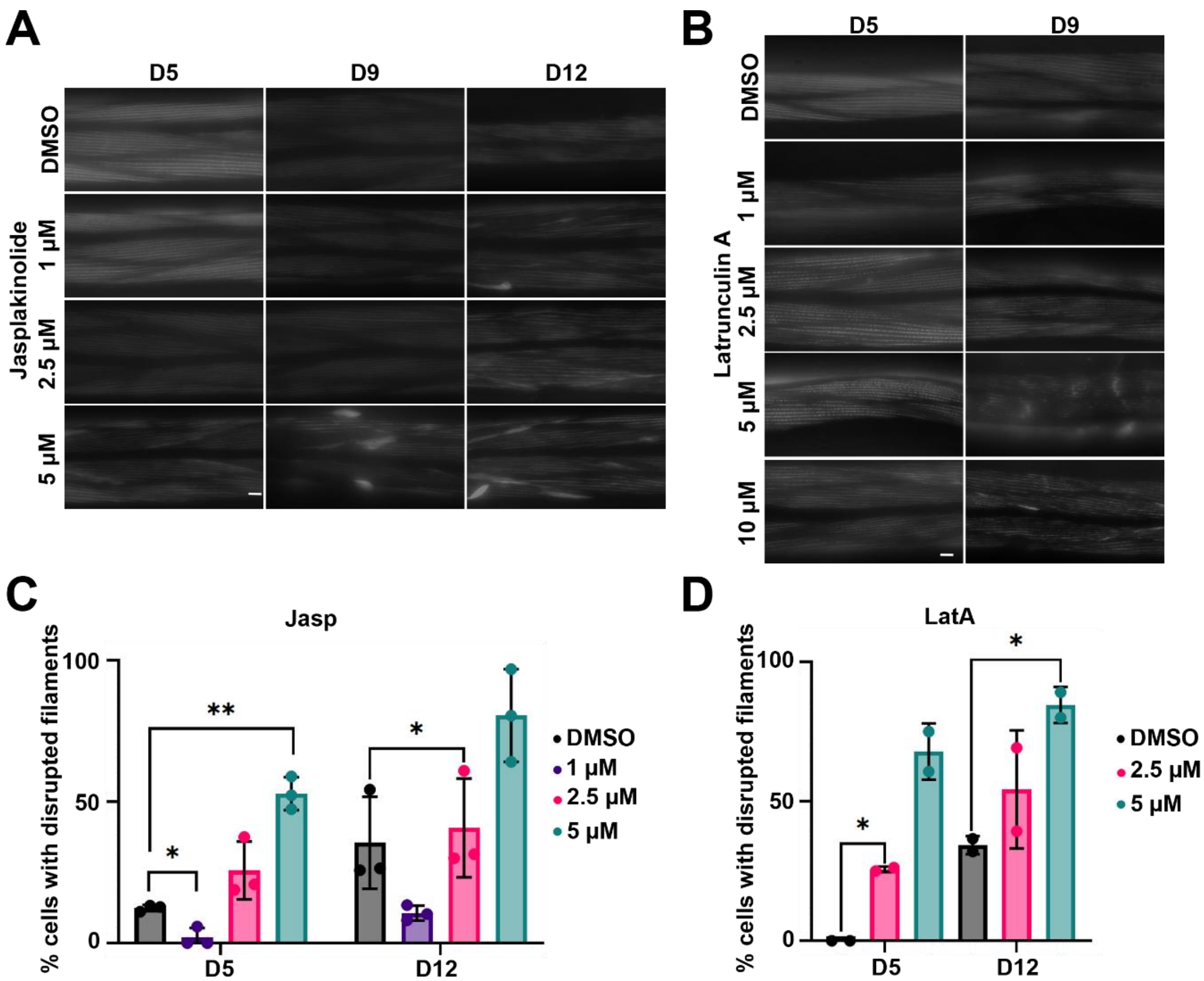

Fig. S13. Small-molecule actin disruption effect on healthspan. (A) Representative fluorescent images of adult animals expressing LifeAct::mRuby in the muscle grown on empty vector (EV) and varying concentrations of Jasplakinolide (1µM, 2.5 µM, 5 µM) or DMSO control. **(B)** Representative fluorescent images of adult animals expressing LifeAct::mRuby in the muscle grown on empty vector (EV) and varying concentrations of Latrunculin A (1µM, 2.5 µM, 5 µM, 10 µM) or DMSO control. All muscle images were captured on day 5, and 9 of adulthood and on a Leica Thunder Imager. **(C-D)** Quantification of actin structure quality in animals expressing LifeAct::mRuby in the muscle grown on empty vector (EV) and varying concentrations of **(C)** Jasplakinolide and **(D)** Latrunculin A (1µM, 2.5 µM, 5 µM) or DMSO control. n=3 N>5.

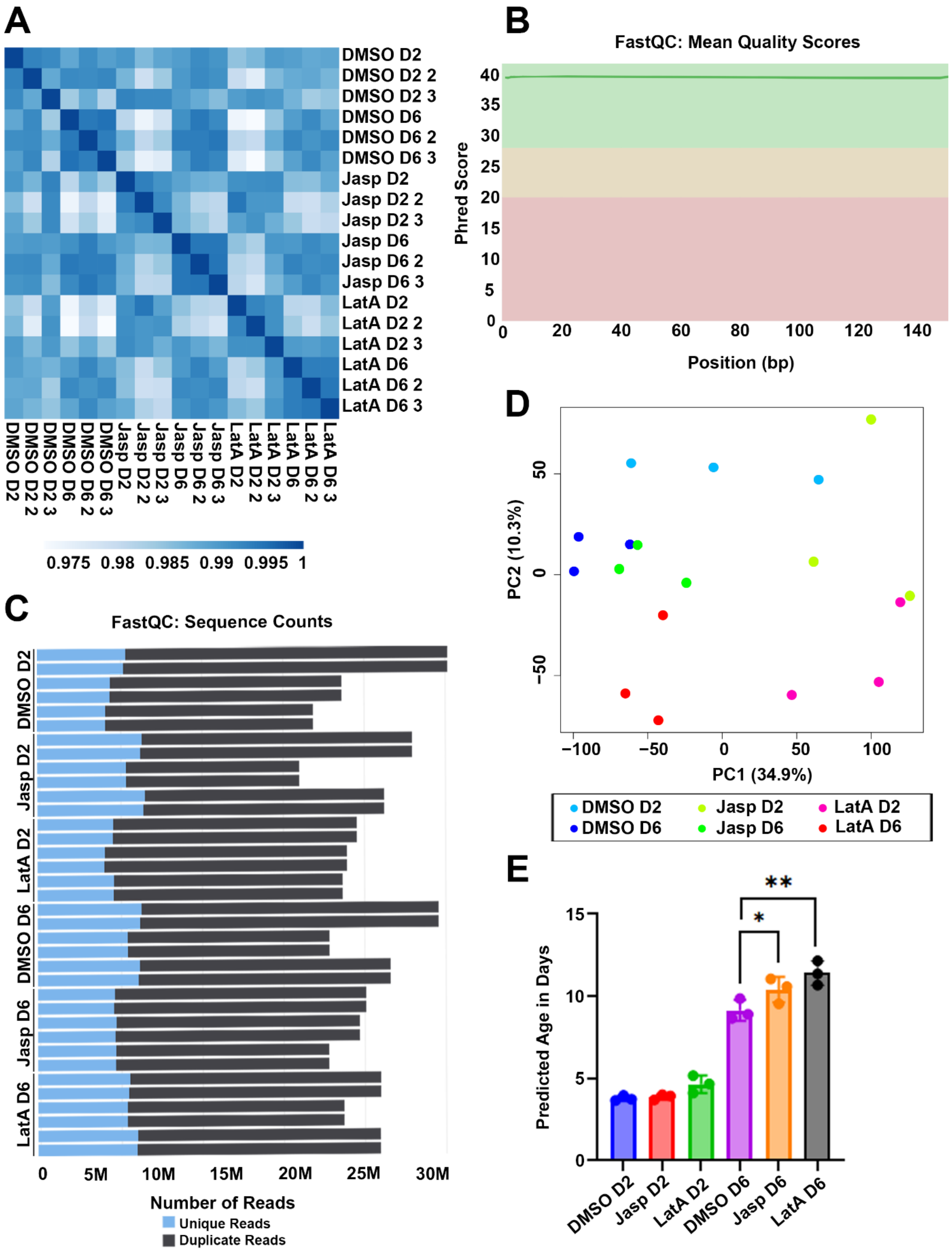

Fig. S14. Quality control of LatA and Jasp RNA-seq data. (A) Spearman correlation plot of all RNA sequencing libraries. **(B)** Mean quality score (Phred score) of each sequencing library.

X-axis and Y-axis indicate the base pair position of each sequence and the Phred score, respectively. The graph was generated by MultiQC tool[199]. **(C)** The number of unique (blue) and duplicated (grey) reads from each pair-wise sequencing library (n=3). **(D)** PCA plots of Jasplakinolide and Latrunculin A exposure in C. elegans. **(E)** Predicted Age of day 2 and day 6 adult worms, using BiT Age algorithm. * = $p < 0.05$, ** = $p < 0.01$ calculated using paired t-test. . See **Table S4** for the BiTAge output.

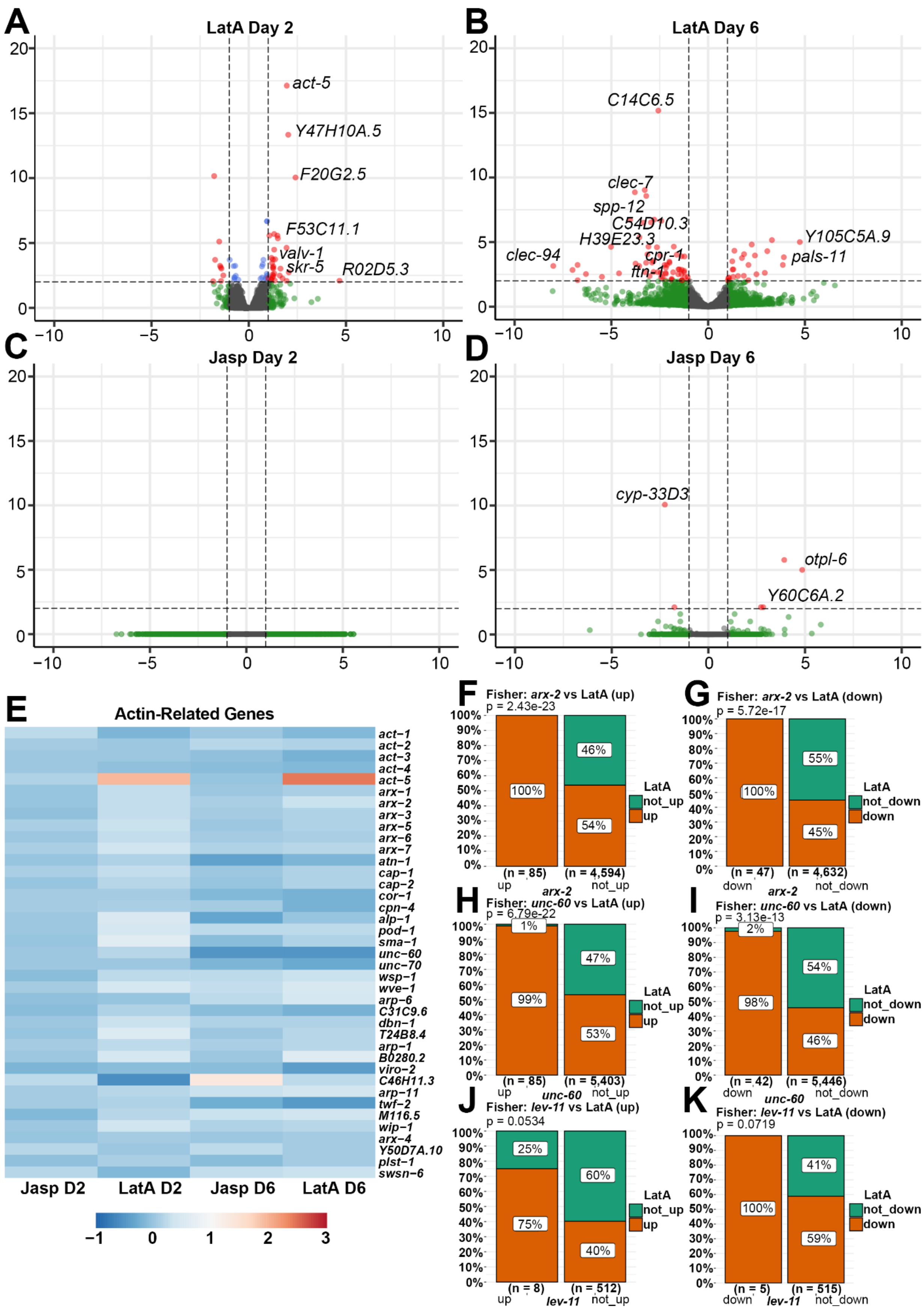
A
LatA Day 2
act-5
Y47H10A.5
F20G2.5
F53C11.1
valv-1
skr-5
R02D5.3
B
LatA Day 6
C14C6.5
clec-7
spp-12
C54D10.3
H39E23.3
clec-94
cpr-1
ftn-1
Y105C5A.9
pals-11
C
Jasp Day 2
D
Jasp Day 6
cyp-33D3
otpl-6
Y60C6A.2
E
Actin-Related Genes
act−1
act−2
act−3
act−4
act−5
arx−1
arx−2
arx−3
arx−5
arx−6
arx−7
atn−1
cap−1
cap−2
cor−1
cpn−4
alp−1
pod−1
sma−1
unc−60
unc−70
wsp−1
wve−1
arp−6
C31C9.6
dbn−1
T24B8.4
arp−1
B0280.2
viro−2
C46H11.3
arp−11
twf−2
M116.5
wip−1
arx−4
Y50D7A.10
plst−1
swsn−6
Jasp D2
LatA D2
Jasp D6
LatA D6
F
Fisher: arx-2 vs LatA (up)
p = 2.43e-23
G
Fisher: arx-2 vs LatA (down)
p = 5.72e-17
H
Fisher: unc-60 vs LatA (up)
p = 6.79e-22
I
Fisher: unc-60 vs LatA (down)
p = 3.13e-13
J
Fisher: lev-11 vs LatA (up)
p = 0.0534
K
Fisher: lev-11 vs LatA (down)
p = 0.0719
LatA
not_up
up
not_down
down
(n = 85)
(n = 4,594)
(n = 47)
(n = 4,632)
(n = 85)
(n = 5,403)
(n = 42)
(n = 5,446)
(n = 8)
(n = 512)
(n = 5)
(n = 515)
arx-2
unc-60
lev-11

Fig. S15. Actin-binding small molecules induces a mild transcriptional response. Volcano plots of genome-wide changes in gene expression upon drug exposure from Day 1 of adulthood on LatA until **(A)** Day 2 and **(B)** Day 6, and Jasp until **(C)** Day 2 and **(D)** Day 6 of adulthood. Red dots indicate significantly differentially expressed genes with p-value ≤ 0.01 and $\log_2$FC>|2|. Blue dots indicate significantly differentially expressed genes with p-value ≤ 0.01 and $\log_2$FC<|2|. Green dots indicate significantly differentially expressed genes with p-value ≥0.01. See **Table S11** for a list of differentially expressed genes and expression values. **(E)** Heat map of differentially expressed genes annotated as "cytoskeleton: Actin function" in WormCat[200]. Warmer colors indicate increased expression, and cooler colors indicate decreased expression. See **Table S6** for expression details of the genes used in the heatmap. **(F-K)** Bar plots to visualize the overlap of up- and down-regulated differentially expressed genes (DEGs) between animals treated with **(F)(G)** *arx-2* and LatA, **(H)(I)** *unc-60* and LatA, and **(J)(K)** *lev-11* and LatA, using ggbarstats function in R. Statistical significance of the overlap was assessed using Fisher's exact test.

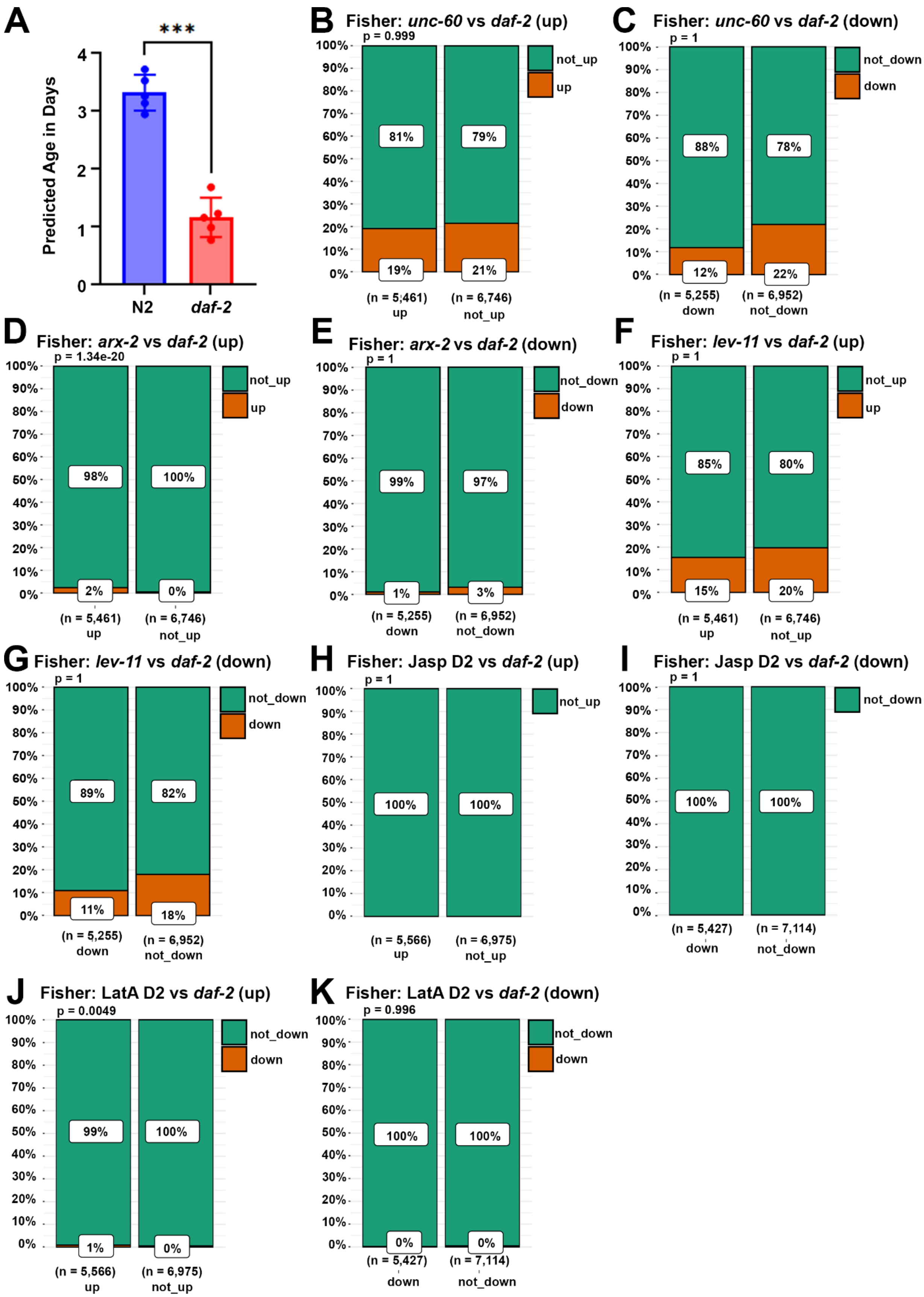

A
***
Predicted Age in Days
4
3
2
1
0
N2
daf-2
B Fisher: unc-60 vs daf-2 (up)
p = 0.999
not_up
up
81%
79%
19%
21%
(n = 5,461) up
(n = 6,746) not_up
C Fisher: unc-60 vs daf-2 (down)
p = 1
not_down
down
88%
78%
12%
22%
(n = 5,255) down
(n = 6,952) not_down
D Fisher: arx-2 vs daf-2 (up)
p = 1.34e-20
not_up
up
98%
100%
2%
0%
(n = 5,461) up
(n = 6,746) not_up
E Fisher: arx-2 vs daf-2 (down)
p = 1
not_down
down
99%
97%
1%
3%
(n = 5,255) down
(n = 6,952) not_down
F Fisher: lev-11 vs daf-2 (up)
p = 1
not_up
up
85%
80%
15%
20%
(n = 5,461) up
(n = 6,746) not_up
G Fisher: lev-11 vs daf-2 (down)
p = 1
not_down
down
89%
82%
11%
18%
(n = 5,255) down
(n = 6,952) not_down
H Fisher: Jasp D2 vs daf-2 (up)
p = 1
not_up
100%
100%
(n = 5,566) up
(n = 6,975) not_up
I Fisher: Jasp D2 vs daf-2 (down)
p = 1
not_down
100%
100%
(n = 5,427) down
(n = 7,114) not_down
J Fisher: LatA D2 vs daf-2 (up)
p = 0.0049
not_down
down
99%
100%
1%
0%
(n = 5,566) up
(n = 6,975) not_up
K Fisher: LatA D2 vs daf-2 (down)
p = 0.996
not_down
down
100%
100%
0%
0%
(n = 5,427) down
(n = 7,114) not_down

Fig. S16. Actin perturbation induces a transcriptional response opposite of the pro-longevity *daf-2* signature. (A) Predicted Age of adult worms grown on control compared to *daf-2* RNAi, using BiT Age algorithm. * = $p < 0.05$, ** = $p < 0.01$ calculated using paired t-test. . See **Table S4** for the BiTAge output **(B-K)** Bar plots to visualize the overlap of up- and down-regulated differentially expressed genes (DEGs) between day 1 adult animals exposed from L1 to RNAi **(B-C)** *unc-60* and *daf-2*, **(D-E)** *arx-2* and *daf-2*, **(F-G)** *lev-11* and *daf-2*, and day 2 adult animals exposed from D1 to **(H-I)** Jasp and *daf-2*, and **(J-K)** LatA and *daf-2* using ggbarstats function in R. Statistical significance of the overlap was assessed using Fisher's exact test.

Supplemental Tables

**RNAi Clone**	**RNAi Sequence**
act-1	TGTGTGACGACGAGGTTGCCGCTCTTGTTGTAGACAATGGATCCGGAA<br>TGTGCAAGGCCGGATTCGCCGGAGACGACGCTCCACGCGCCGTGTTC<br>CCATCCATTGTCGGAAGACCACGTCATCAAGGAGTCATGGTCGGTATG<br>GGACAGAAGGACTCGTACGTCGGAGACGAGGCCCAATCCAAGAGAGG<br>TAAATAATTAATACATTCGATGATTAAATTTATGCGTACTATTTCAGGTA<br>TCCTTACCCTCAAGTACCCAATTGAGCACGGTATCGTCACCAACTGGG<br>ATGATATGGAGAAGATCTGGCATCACACCTTCTACAATGAGCTTCGTGT<br>TGCCCCAGAAGAGCACCCAGTCCTCCTCACTGAAGCCCCACTCAATCC<br>AAAGGCTAACCGTGAAAAGATGACCCAAATCATGTTCGAGACCTTCAA<br>CACCCCAGCCATGTATGTCGCCATCCAAGCTGTCCTCTCCCTCTACGC<br>TTCCGGACGTACCACCGGAGTCGTCCTCGACTCTGGAGATGGTGTCA<br>CCCACACCGTCCCAATCTACGAAGGATATGCCCTCCCACACGCCATCC<br>TCCGTCTTGACTTGGCTGGACGTGATCTTACTGATTACCTCATGAAGAT<br>CCTTACCGAGCGTGGTTACTCTTTCACCACCACCGCTGAGCGTGAAAT<br>CGTCCGTGACATCAAGGAGAAGCTCTGCTACGTCGCCCTCGACTTCGA<br>GCAAGAAATGGCCACCGCCGCTTCTTCCTCTTCCCTCGAGAAGTCCTA<br>CGAACTTCCTGACGGACAAGTCATCACCGTCGGAAACGAACGTTTCCG<br>TTGCCCAGAGGCTATGTTCCAGCCATCCTTCTTGGGTATGGAGTCCGC<br>CGGAATCCACGAGACTTCTTACAACTCCATCATGAAGTGCGACATTGAT<br>ATCCGTAAGGACTTGTACGCCAACACTGTTCTTTCCGGAGGAACCACC<br>ATGTACCCAGGAATTGCTGATCGTATGC
arx-2	TTTCAGGCGAAATGGATTCGCAAGGGCGAAAGGTGATTGTCGTTGACA<br>ACGGAACAGGTGTAAGTTGCAAAATTTTATCCCATTTTATTTTCAAAAAT<br>TCTGTATTTGCAGTTCGTCAAATGCGGGTATGCAGGAACCAATTTCCCA<br>GCTCATATATTCCCTTCAATGGTCGGTCGGCCAATCGTGAGATCGACA<br>CAAAGAGTTGGAAATATTGAAATTAAGGTAAAACTTTACACAGAAAGGT<br>CTTATAACTGTTTTCTCACAGGATTTGATGGTTGGCGAGGAGTGCTCTC<br>AGCTTCGTCAAATGCTTGATATCAATTATCCCATGGACAACGGAATTGT<br>TAGAAACTGGGATGATATGGCGCATGTATGGGATCATACTTTTGGACC<br>GGAAAAGTTGGATATCGACCCGAAAGAGTGTAAACTGCTGCTGACAGA<br>ACCACCACTCAATCCGAACAGCAATCGTGAGAAAATGTTTCAAGTTATG<br>TTTGAGCAGTATGGATTCAATTCTATCTATGTGGCGGTTCAGGTAATAG<br>TTAATCCTCATGATAAAATCATAATGATTATATTTTCAGGCTGTGCTCAC<br>TCTTTATGCTCAAGGTCTTTTGACAGGAGTTGTAGTTGACTCCGGAGAT<br>GGTGTTACCCACATCTGTCCTGTTTACGAAGGATTTGCTCTTCATCATT<br>TGACAAGACGATTGGATATTGCAGGAAGAGATATTACCAAGTATCTTAT<br>CAAGGTAAGTTTTTAAATGTTACATATTAATTCAATTATTTTCCTGATTGC

	TATAGTGACAAAATATGGTTTATTAGAAACTAACATATATTTGTTTTCAG<br>CTTCTTCTGCAACGTGGATACAACTTCAATCACTCTGCTGACTTTGAAA<br>C
lev-11	TGTCGAAGGTAAACAAGGAGGGAGCTCAGCAGACATCGCTTCTTGATG<br>TCCTCAAGAAGAAGATGCGTCAGGCCCGTGAGGAGGCTGAGGCCGCC<br>AAGGACGAGGCCGACGAGGTCAAAAGACAGCTCGAGGAGGAGCGCA<br>AGAAGCGTGAAGATGTATGTGATTATAGTAGCGACGACTAGGTTTCTT<br>GTGATAAGTCTCAATAATTCCATCTAGTGTAACAAGCCCCGCGCGGGC<br>ACTAAGTCAATATAAGTTGTATAAAGTGGCTGCTCCTGCTGCTTATTTG<br>TCTTAGAATAGGAAAAAGTGATGCGAGAGAAAGTGAATGAGGCTATGG<br>GATTTTGTAGGCTGAAGCCGAGGTTGCCGCCTTGAACCGTCGTATTGT<br>GCTTGTGGAGGAGGACTTGGAAAGAACCGAAGATCGTCTGAAGACCG<br>CCACATCAAAGCTTGAACAAGCAACCAAAGCTGCCGATGAGGCTGATC<br>GGTTCGTTTTTTTCTATCCCATTTGTGTCAATTACAGGCTGAGGCTGAG<br>GTCGCTTCTTTGAACCGTCGCATGACCCTCCTCGAGGAAGAGCTCGAG<br>AGAGCCGAGGAGCGTCTGAAGATCGCCACCGAGAAGCTCGAAGAGGC<br>TACCCACAATGTCGACGAGTCTGAGCGGTAGGCGTTGAGATGAGGATT<br>CCTGTGGATCTCAAGCCAAACTACCAAACTACTGCCAGTGCTCAGAAA<br>ATTATATATATATTGTGAACGTCATTGTCCCAATTGTGTACTGTAAATTT<br>TTTAGCGCGCGCAAGTCGATGGAAACCCGCTCCCAACAGGACGAGGA<br>ACGTGCCAACTTCCTCGAGACTCAAGTCGACGAGGCTAAGGTTATCGC<br>CGAGGATGCTGATCGCAAATACGAAGAGGTGCCGATTGAATGCGCGT<br>GCTTAATGGCTTAATGGCTTAATGTTAACTGTCATGTGTCCTGAATGTT<br>AAACCCCCCTAATAGTTTGGAGTTTTTTTTTTACAAAATCTAATCTCTAT<br>TATAGATCCAGAAGAGCGTTGAGCAATCAGATTGACATGGATGATGAC<br>AGATGTTCGGATCTTGAAAGGAAGTTAAGAGAGTGTCAGTCGATTTTG<br>CATGAAACTGAAAACAAAGCAGAAGAGGTAGAGCTTTATTTACTTTCAA<br>CTCCTAGAAATTCCATGTTCTAATGCTTTATGTTGTCCGAGTTGTCCGA<br>ACACTGGCACAAGAATACAATGGCACAGAGATCCTATCAGGCGATCCC<br>TCACCCCGAAACTTTTGTTGAATAATACTGTTCCAGCGTGCGCAAGGTT<br>ATGGAGAACCGCTCCCTTCAGGATGAGGAGCGCGCCAACACCGTTGA<br>GGCCCAACTTAAGGAGGCTCAACTTCTCGCCGAGGAGGCTGACCGCA<br>AATACGACGAGGTTACCATCGAGTGGATTATATATATATATATTATATAA<br>TCATATAACATGTTCTCATACTAATTGTCTTGCATCTTTAACACTATAAC<br>ATTAGACGTATGTGACAAAAATAATAATGAAAATTGTTCCAGGTCGCCC<br>GTAAGCTCGCCATGGTTGAAGCTGATCTTGAGAGAGCTGAGGAGCGT<br>GCCGAGGCCGGAGAGAAGTGAGTTAAGAGCACCTATCACAATGCCCC<br>CTTTTCATTTACTTTTACGCTTCTTAACCCAACTAACAAAAACGCATAAT<br>TCTAATAAAACCGATAAAACATAAAACCACCATCACAGCAAGATCGTGG<br>AGCTTGAAGAGGAGTTGCGCGTCGTTGGTAATAACTTGAAATCCCTTG<br>AACTTTCCGAGGAAAAGGCACTTGAGAAGGAGGACATCTTTGCCGAGC<br>AGATTCGTCAGCTCGATTTCAGACTGAAGGAGGTATCAACGTGTCTCA<br>GAAAGTCTCTCTTCACCTCAAAACCTTCCGGAATACCTTTTGAGATGAT<br>ATGCTTATTAACAAACTCATTCGCTTTAACAATCAAGCTTCAAACTTTGT<br>ATTCCTCTAATCTTAACTTGGGTGACATTCTTAGCCTAACCTTACAGAC<br>GATCTAACCTGGCAGAGGCCCACATGCGCGGGCTCTCCGTGAATCTT<br>CGTGAGGCGCAGGACCTGTTACATCAGCTGCAGCAGGAGGAGAACGA<br>TTCCTGCGAATACTTGAATTGCGCCGTAGAATCGCGAAAGGAGGTTTC<br>TCAAACACCATTTGTTCATAAAACTAATGCTTCTGCACGACACTAAACT<br>CTTACTCATTCCCATCTTCACATCTCTCTCTCTCCCCCTTTCAAACCGTC<br>GTTGTCCCATCTTTCAGCAAGATCGTCGAACTCGAGGAGGAGCTCCGT

	GTCGTCGGAAACAACTTGAAGTCACTTGAGGTGTCCGAGGAGAAGGCT<br>CTCCAACGTGAGGACTCGTACGAGGAGCAGATCCGCACCGTCTCATC<br>CAGACTGAAGGAGGTCCGATTCTTTTCTGTGTAGTAGTCTTTCTGTGTC<br>TTCACTATCAAAGTGATGTCCGTGTTGTCTTTAATTGTGATAATAGTTCC<br>TGAAAAAAAAACGAAATTTCTTTCAGGCTGAGACCCGTGCCGAATTCG<br>CTGAGCGTTCCGTCCAGAAGCTCCAGAAGGAGGTCGACAGACTCGAA<br>GGTAGGTCTAAGGGGCTTTATATGTGATATCTAAATGTCATTGTCAATT<br>TGTGTAAGTCTATATGTGTATTGTCCACGTGTTCAGATGAACTCCTCCT<br>CGAGAAGGAGCGTGTCCGAAACTTGACTGAAGAAATCGAACAGACCGT<br>CCAGGAGATCCAAGGATCCTA
unc-60	ATGGTGAGTTTGAGATTTTAATTCGCTTCAATATTTTTAAATTGAAAGAA<br>ATTTGGAATGTTATGTTTGGCGTGTTTTTTTTTCATTGAATTTCCAAAATT<br>TCACACTAAAATGGAATGGTTTTCTCTTCTTTTTGCCTAACCTAACCTAA<br>CCTATGTGTGCCTGTTTTCTAGAGTTCCGGTGTCATGGTCGACCCAGA<br>TGTGCAGACATCTTTCCAAAAGCTCTCCGAGGGACGCAAGGAGTACCG<br>CTACATCATTTTCAAGATCGACGTGAGTTTTTAAAAAATAAAATCTGAAT<br>CAGATCAATTTAAAAAAATTTCGTGCCACTTTTTGTTTTTTTTGTTGAAAA<br>ATTTGAAAAATCCTCAAATTAATTGTTTTAGATGAACATTATTGATTTCC<br>CTATTAAATTGCAACATTTTCCAGGAGAACAAGGTGATCGTGGAAGCC<br>GCGGTGACTCAGGATCAGCTCGGCATCACTGGAGACGACTATGATGA<br>CTCTTCCAAGGCCGCTTTCGACAAATTCGTCGAGGACGTGAAGTCTCG<br>AACCGATAATCTGACCGATTGCCGCTACGCCGTTTTCGACTTCAAGTTC<br>ACGTGCAGTCGTGTTGGAGCCGGCACGAGCAAGATGGACAAGATCAT<br>CTTCCTCCAGATGTAAGCGCTTGATCCTATTGGTGAATTATTGTACCAT<br>CTAATTTTTTTTCCAGCTGCCCAGATGGTGCTTCTATCAAGAAAAAGAT<br>GGTGTACGCTTCGTCCGCCGCCGCCATCAAGACTTCTCTCGGAACCG<br>GCAAAATCCTTCAGTTCCAGGTGAGAAATCTCGATAATTTTTACAATTA<br>GAAAAAAAAAATCAAATTATTAAAATTTCAGGTGTCTGACGAGAGCGAG<br>ATGAGCCACAAGGAACTCCTCAACAAGTTGGGCGAGAAATACGGAGAT<br>CACTAG

Table S12. RNAi sequences.

Strains used in this study		
C. elegans: Bristol (N2) strain as wild type (WT)	CGC	N2
C. elegans: SS104: glp-4(bn2) I	CGC	SS104
C. elegans: ML2615: dlg-1(mc103[dlg-1::gfp]) X	[75]CGC	ML2615
C. elegans: RHS41: unc-119(ed3) III; uthSi7[myo-3p::LifeAct::mRuby::unc-54 3'UTR::cb-unc-119(+)] IV	[2]CGC	AGD1651 backcrossed 4x
C. elegans: RHS42: unc-119(ed3) III; uthSi10[col-19::LifeAct::mRuby::unc-54 3'UTR::cb-unc-119(+)] IV	[2]CGC	AGD1654 backcrossed 4x
C. elegans: RHS43: uthSi13[gly-19p::LifeAct::mRuby::unc-54 3'UTR, cb-unc-119(+)]	[2]CGC	AGD1657 backcrossed 4x
C. elegans: AGD1395: uthIs393[vha-6p::Q40::YFP+rol-6(su1006)]	[153]Dillin lab	AGD1395

C. elegans: MAH602: sqIs61[vha-6p::Q44::YFP + rol-6(su1006)]	[71]Hansen Lab	MAH602
C. elegans: RHS80: rmIs110[rgef-1p::Q40::YFP]	[64]CGC	AM101 backcrossed 7x
C. elegans: RHS75: rmIs133[unc-54p::Q40::YFP]	CGC	AM141 backcrossed 8x
C. elegans: MAH349: sqIs35[pMH951/sqst-1p::sqst-1::gfp+pMH876/unc-122p::rfp]	[71]CGC	MAH349
C. elegans: MAH1192: atg-3(bp412) IV ;sqIs35[sqst-1p::sqst-1::gfp+unc-122p::rfp]	Hansen Lab	MAH1192
C. elegans: DA2123: adIs2122[lgg-1p::GFP::lgg-1 + rol-6(su1006)]	[154]CGC	DA2123
C. elegans: RHS191: uthSi17[myo-3p::MLS::GFP(65C)::unc-54 3'UTR, cb-unc-119(+)] I;	[155]CGC	AGD1664 backcrossed 4x
C. elegans: RHS192: uthSi83[col-19p::MLS::GFP(65C)::unc-54 3'UTR, cb-unc-119(+)] I;	[155]CGC	AGD2837 backcrossed 4x
C. elegans: RHS193: uthSi80[vha-6p::MLS::GFP(65C)::unc-54 3'UTR, cb-unc-119(+)] IV;	[155]CGC	AGD2805 backcrossed 4x
C. elegans: AM140: rmIs132[unc-54p::Q35::YFP];	[156]CGC	AM140
C. elegans: NR222: rde-1(ne219) V; kzIs9[(pKK1260) lin-26p::NLS::GFP + (pKK1253) lin-26p::rde-1 + rol-6(su1006)]	[157]CGC	NR222
C. elegans: VP303: rde-1(ne219) V; kbIs7[nhx-2p::rde-1 + rol-6(su1006)]	[158]CGC	VP303
C. elegans: WM118: rde-1(ne300) V; neIs9[myo-3::HA::RDE-1 + rol-6(su1006)] X	[159]CGC	WM118
C. elegans: MAH677: sid-1(qt9) V; sqIs71[rgef-1p::GFP + rgef-1p::sid-1]	[133]CGC	MAH677
C. elegans: RHS10: ldrIs1[dhs-3p::dhs-3::GFP + unc-76(+)]	[152]	AGD2048 backcrossed 4x

Table S13. Strains used in this study.
