# Supplementary material for: Form and function of actin impacts actin health and aging
Source: iScience. 2026 Aug 24;29(9):117256. doi: 10.1016/j.isci.2026.117256 (PMC13524715; doi:10.1016/j.isci.2026.117256)
Supplement: Supplementary file 2 [file mmc13.pdf]

A.) .IJM code for hypodermal actin quantification

```
run("Clear Results"); // clear the results table of any previous measurements
setBatchMode(true);
inputDirectory = getDirectory("Choose a Directory of Images");
fileList = getFileList(inputDirectory);
for (i = 0; i < fileList.length; i++)
{
   processImage(fileList[i]);
}
setBatchMode(false); // Now disable BatchMode since we are finished

updateResults();  // Update the results table so it shows the filenames

// Show a dialog to allow user to save the results file
outputFile = File.openDialog("Save results file");
// Save the results data
saveAs("results",outputFile);

function processImage(imageFile)
{
         // Store the number of results before executing the commands,
   // so we can add the filename just to the new results
         prevNumResults = nResults;
         open(imageFile);
   // Get the filename from the title of the image that's open for adding to the results table
   // We do this instead of using the imageFile parameter so that the
   // directory path is not included on the table
   filename = getTitle();
         run("8-bit");
         setAutoThreshold("Default dark no-reset");
         run("Convert to Mask");
         run("Set Measurements...", "area mean min area_fraction redirect=None decimal=3");
         run("Measure");

            for (row = prevNumResults; row < nResults; row++)
         {
      setResult("Filename", row, filename);
   }

   close("*");  // Closes all images
}
```

B.) .IJM code for lipid droplet quantification

```
// Close existing Results table if open
if (isOpen("Results")) {
    selectWindow("Results");
    run("Close");
}

// Prepare a dummy measurement to initialize Results table
run("Set Measurements...", "area mean integrated redirect=None decimal=3");
run("Measure");
setResult("Label", 0, "Init");
updateResults();
run("Clear Results");

for (img = 0; img < nImages; img++) {
    selectImage(img + 1);
    title = getTitle();

    // Switch to channel 2
    Stack.setChannel(2);
    run("8-bit");
    run("Enhance Contrast", "saturated=0.35");

    // Prompt for ROI input
    roiManager("reset");
    waitForUser("Image: " + title + " (Channel 2)\nDraw ROIs around each worm and add them to
the ROI Manager.\nClick OK to continue.");

    nWorms = roiManager("count");

    for (i = 0; i < nWorms; i++) {
        roiManager("Select", i);
        run("Measure");

        // Ensure the Results table is selected before editing
        selectWindow("Results");
        currentRow = nResults - 1;
        label = "Worm_" + (i+1) + "_" + title;
        setResult("Label", currentRow, label);
        updateResults();
    }
}

// Ask user where to save the file
saveDir = getDirectory("Choose a folder to save your CSV");
```

```
filePath = saveDir + "IntegratedIntensity_Summary_sqstR3.csv";

// Save the results
selectWindow("Results");
saveAs("Results", filePath);
```

C.).IJM code for gut assay quantification

```
// Close existing Results table if open
if (isOpen("Results")) {
    selectWindow("Results");
    run("Close");
}

// Prepare a dummy measurement to initialize Results table
run("Set Measurements...", "area mean integrated limit  redirect=None decimal=3");
run("Measure");
setResult("Label", 0, "Init");
updateResults();
run("Clear Results");

for (img = 0; img < nImages; img++) {
    selectImage(img + 1);
    title = getTitle();

    // Switch to channel 2
    Stack.setChannel(2);
    run("8-bit");
   // run("Enhance Contrast", "saturated=0.35");

    // Prompt for ROI input
    roiManager("reset");
    waitForUser("Image: " + title + " (Channel 2)\nDraw ROIs around each worm and add them to
the ROI Manager.\nClick OK to continue.");

    nWorms = roiManager("count");

    for (i = 0; i < nWorms; i++) {
        roiManager("Select", i);
        Stack.setChannel(2);
        setThreshold(25, 255); // Only include pixels above 25
        setOption("BlackBackground", true);
```

```
        run("Measure");

        // Ensure the Results table is selected before editing
        selectWindow("Results");
        currentRow = nResults - 1;
        label = "Worm_" + (i+1) + "_" + title;
        setResult("Label", currentRow, label);
        updateResults();
    }
}

// Ask user where to save the file
saveDir = getDirectory("Choose a folder to save your CSV");
filePath = saveDir + "dayx_replicatex _IntegratedIntensity_Summary_gut_assay.csv";

// Save the results
selectWindow("Results");
saveAs("Results", filePath);
```

D.) .IJM code for lipid droplet and neuronal puncta quantification

```
if (isOpen("Summary")) { selectWindow("Summary"); run("Close"); }
for (img = 0; img < nImages; img++) {
    selectImage(img + 1);
    title = getTitle();

    // Switch to channel 3
    Stack.setChannel(3);

    // Prompt user to draw worm ROIs
    roiManager("reset");
    waitForUser("Image: " + title + " (Channel 3)  \nDraw ROIs around each worm and add them
to the ROI Manager.\nClick OK to continue.");

    nWorms = roiManager("count");

    for (i = 0; i < nWorms; i++) {
        // Select ROI and duplicate region
        roiManager("Select", i);
        newTitle = "Worm_" + (i+1) + "_" + title;
        run("Duplicate...", "title=" + newTitle);
```

```
        actualTitle = getTitle();
        selectWindow(actualTitle);

        // Reapply ROI before clearing
        roiManager("Select", i);
        run("Clear Outside");

        run("8-bit");
        run("Subtract Background...", "rolling=30");
        run("Auto Threshold", "method=MaxEntropy");
        setOption("BlackBackground", true);
        run("Convert to Mask");
        run("Erode");
        run("Dilate");
                //waitForUser("Review thresholding for " + newTitle + ". Zoom/pan if needed, then
click OK to continue.");

        // Analyze particles into summary table with custom name
        run("Set Measurements...", "area mean integrated redirect=None decimal=3");
        run("Analyze Particles...", "size=0-Infinity circularity=0.0-1.00 summarize");

        // Rename the Slice label in Summary table
        sliceName = "Worm_" + (i+1) + "_" + title;
        Table.set("Slice", Table.size - 1, sliceName);

        close();
    }
}

// Save the summary table to a folder of your choice
saveDir = getDirectory("Choose a folder to save your CSV");
filePath = saveDir + "Counts_SummaryTable.csv";
selectWindow("Summary");
saveAs("Results", filePath);
```
